# Genotypic variability in stress responses of Sorghum bicolor under drought and salinity conditions

**DOI:** 10.3389/fgene.2024.1502900

**Published:** 2025-01-08

**Authors:** Yahya Alzahrani, Abdulbaki Shehu Abdulbaki, Hameed Alsamadany

**Affiliations:** ^1^ Department of Biological Sciences, Faculty of Science, King Abdulaziz University, Jeddah, Saudi Arabia; ^2^ Department of Plant Science and Biotechnology, Faculty of Life Sciences, Federal University Dutsinma, Dutsinma, Katsina State, Nigeria

**Keywords:** Sorghum bicolor, abiotic resilience, genetic variation, oxidative stress mitigation, sustainable agriculture, targeted breeding

## Abstract

**Introduction:**

Sorghum bicolor: widely cultivated in Asia and Africa, faces increasing challenges from climate change, specifically from abiotic stresses like drought and salinity. This study evaluates how different sorghum genotypes respond to separate and combined stresses of drought and salinity.

**Methods:**

Carried out with three replications using a randomized complete block design, the experiment measured biochemical and physiological parameters, including stomatal conductance, chlorophyll content, and antioxidant enzyme activities. Molecular analysis focused on stress-responsive gene expression.

**Results:**

Results indicated enhanced stress responses under combined conditions, with significant variation in antioxidant enzymatic activities among genotypes. Genotype-specific osmotic adjustments were observed through proline and glycine betaine accumulation. Physiological parameters such as chlorophyll content, cell membrane stability, stomatal conductance, and water potential were critical indicators of stress tolerance. Gene expression analysis revealed upregulation of stress-responsive genes, particularly under combined stress conditions.

**Discussion:**

Correlation and principal component analysis analyses highlighted the interdependencies among traits, emphasizing their roles in oxidative stress mitigation. Samsorg-17 exhibited the highest resilience due to consistently high levels of catalase, superoxide dismutase, and glycine betaine, alongside superior physiological attributes. CRS-01 showed moderate resilience with the highest Na/K ratio and notable photosynthesis rate and relative water content, but was less consistent in biochemical markers under stress. Samsorg-42 demonstrated resilience under specific conditions but was generally less robust than Samsorg-17 across most indicators. These findings emphasize the importance of developing stress-resilient sorghum cultivars through targeted breeding programs to enhance tolerance to drought and salinity in sustainable agriculture.

## 1 Introduction

*Sorghum bicolor* (L. Moench) is the cultivated species of the genus Sorghum. It is commonly called sorghum or great millet. It is a cereal grown widely around the world and particularly in the Asia and Africa continents (Almaiman et al., 2021). Ranked the fifth most important crop globally (ICRISAT, 2016; Balakrishna et al., 2018), sorghum is renowned for its adaptability to diverse environmental conditions (Yahaya et al., 2023). However, the escalating impacts of climate change have amplified the occurrence and severity of abiotic stresses, posing significant challenges to sorghum production worldwide (Druille et al., 2020; Chadalavada et al., 2021). Among these stressors, drought and salinity are two of the most pervasive and detrimental factors that impede crop growth and yield. As sorghum is often cultivated in regions prone to water scarcity and soil salinization, understanding its responses to these simultaneous stresses is paramount for sustainable agriculture.

When plants experience drought, salt, or combined stress conditions, they undergo various physiological and biochemical changes. For instance, in a study on pepper cultivars, researchers observed decreased photosynthetic activity, stomatal conductivity, and transpiration rate under both stressors (Yildirim et al., 2022; Abdulbaki et al., 2024). Antioxidant enzyme activity, proline, and sugar content changed as adaptive responses (Yildirim et al., 2022). Transcript profiling studies have shown that gene expression is altered in response to drought and salt stress (Ghorbani et al., 2019). Additionally, combined salinity-drought stress has a greater negative impact on plant growth, photosynthesis, ionic balance, and oxidative balance than either stress alone. Biochemical traits, such as phytohormone content and non-structural carbohydrates, also play a crucial role in stress adaptation (Li et al., 2024).

Although sorghum is considered tolerant to drought since it can survive in many drought-prone fields, the effect of water deficit is still felt in its growth and development (Hadebe et al., 2017). In fact, drought is the topmost abiotic stress that affects its production (Assefa et al., 2010). However, sorghum is still better adapted to drought than other C4 cereals (Amaducci et al., 2016). The impact of water stress on sorghum cut across all its growth stages; from germination to reproductive and grain filling stage (Kapanigowda et al., 2013; Rana et al., 2017; Sehgal et al., 2018; Queiroz et al., 2019).

Equally, sorghum is averagely salt-tolerant. When sorghum is exposed to high salt concentrations, there is perceived reduction in many morpho-physiological parameters (Netondo et al., 2004; Kafi et al., 2011; Roy et al., 2018). The performance of sorghum genotypes in response to salinity during the seedling stage is an important indicator for identifying salt-tolerant varieties. This has been validated through testing 10 different genotypes (Dehnavi et al., 2020).

Consequently, the significance of specific stress-responsive genes in sorghum’s adaptive mechanisms cannot be overstated. Genes such as SbSOD1, SbAPX2, and SbCAT3 play crucial roles in the antioxidant defense system, mitigating oxidative damage by scavenging reactive oxygen species (Pant and Huang, 2021; Zhang et al., 2023). SbHKT1; 4 is involved in ion homeostasis, helping to maintain cellular ion balance under salinity stress (Guo et al., 2020). The transcription factors SbDREB2A and SbDHN3 are pivotal in regulating gene expression in response to drought and salinity, enhancing stress tolerance (Nagaraju et al., 2018; Singh and Chandra, 2021).

SbPRP1 contributes to maintaining cell wall integrity under stress conditions (Rajasheker et al., 2022). Understanding the functions of these genes helps elucidate the molecular pathways sorghum employs to withstand adverse environmental conditions.

Abiotic stress tolerance is crucial for sustaining crop productivity and ensuring global food availability amidst environmental challenges. Developing resistant genotypes is a key strategy to enhance tolerance to these stresses (Olayinka et al., 2021). By focusing on the genetic and physiological mechanisms underlying stress responses, researchers can identify traits that confer resilience and use them in breeding programs to produce robust, high-yielding cultivars. This approach not only improves crop performance under adverse conditions but also supports sustainable agricultural practices by reducing the need for inputs like water and fertilizers.

The primary aim of the present research is to deepen our understanding of sorghum’s adaptive mechanisms to combined abiotic stresses, particularly drought and salinity, by integrating molecular, physiological, and biochemical perspectives. The study therefore seeks to unravel the complex regulatory networks, key genes, and pathways that underpin sorghum’s resilience to these stressors, ultimately contributing to the development of stress-tolerant cultivars for sustainable agriculture in the face of climate change. The current investigation enhances our knowledge of sorghum’s resilience to various stressors and offers valuable insights for developing stress-tolerant sorghum cultivars and sustainable agricultural practices amidst climate change-induced challenges.

## 2 Materials and methods

### 2.1 Treatment application and description of the sorghum cultivars

The sorghum cultivar seeds utilized in the present study are high-yielding and were obtained from the Institute for Agricultural Research (IAR) in Nigeria. The names of the cultivars are Samsorg-17, Samsorg-42 and CRS-01. The study was carried out in the experimental area of the Biological Sciences Department at King Abdulaziz University in Jeddah, Saudi Arabia (21.4999°N, 39.2334°E). Under controlled environment of a glass house, it utilized 20 kg porcelain pots (with 30 cm diameter) loaded with a mixture of 15 kg peat moss and soil. The soil contained slightly alkaline (pH 8.3) loam.

The research utilized a randomized complete block pattern with a tripartite replication. Details of the treatments included: D1- 10 days withholding irrigation, D1S1- 10 days and 200 mM NaCl, D1S2- 10 days and 300 mM NaCl, D2- 20 days withholding irrigation, D2S1- 20 days and 200 mM NaCl, D2S2- 20 days and 300 mM NaCl, S1- 200 mM NaCl, S2- 300 mM NaCl and C-control. Salt stress was induced on a weekly basis by treating the soil with 100 mM and 200 mM NaCl. Readings were recorded following the imposition of stress at three-leaf stage of growth. The drought treatment durations were based on previous studies imposing drought stress for different periods in sorghum and barley between 2 and 3 weeks (Gao et al., 2020; Romdhane et al., 2020; Yang et al., 2020; Abreha et al., 2021). Similarly, the choice of 200–300 mM NaCl concentrations for inducing salinity stress was informed by earlier research on barley and sorghum (Huang, 2018; Zhu et al., 2020; Dwevedi, 2021).

### 2.2 Collection of data

#### 2.2.1 Biochemical parameters

The glycine betaine levels were measured using a spectrophotometric method involving its reaction with iodine, following the procedure outlined by Valadez-Bustos et al. (2016). Using a spectrophotometer, Proline was also analyzed using a similar method involving its reaction with ninhydrin.

In order to assess the activities of antioxidant enzymes such as catalase (CAT), superoxide dismutase (SOD) and peroxidase (POD), the technique outlined by Djanaguiraman et al. (2014) was adopted. 2 g of homogenous, frozen leaf samples were combined with 2 mL of (ice-cold) 0.1 M Tris-HCl buffer. The mixture was then centrifuged for 15 min at 4°C and 2000 rpm. The resulting supernatant was collected, and the activity of the enzymes was assayed using specific assay kits–CAT and SOD assay kits from Sigma-Aldrich and POD assay kit from BiolabsInc–as per the manufacturers’ protocols. Additionally, the antioxidant enzyme activity was quantified as mg^−1^ protein, determined through their respective absorbance standard curves.

The procedure to evaluate lipid peroxidation via MDA content includes the reaction of thiobarbituric acid (TBA) with malondialdehyde (MDA) to form a pinkish chromogen measurable with spectrophotometry.

The MDA content was expressed as nmol per mg protein following its determination using a standard curve (Reilly and Aust, 1999). The level of the superoxide anion radical (O₂⁻) was determined following the process described by Ajiboye et al. (2016). Furthermore, the measurement of hydrogen peroxide (H₂O₂) concentration was conducted adopting the procedure described by Velikova et al. (2000).

#### 2.2.2 Physiological parameters

Stomatal conductance (gs) and photosynthesis rate (pn) were measured spontaneously using the updated CIRAS-3 tool (Amesbury, MA 01913, United States), by placing it on fully expanded leaves. Solute and water potentials were recorded following the method described by Sattar et al. (2020). The Na/K content in the leaves was measured according to the method outlined by Hniličková et al. (2019). Similarly, the leaf chlorophyll content was determined using the procedure outlined by Mahmood et al. (2016). This was achieved by shaking 0.5 g of fresh leaf samples in 80% acetone until the leaves became colorless. The subsequent extract was then centrifuged for 10 min at 13 × 10^3^ rpm, and the supernatant was employed to determine the chlorophyll content (a and b) through spectrophotometeric readings at 663 nm and 645 nm respectively.

The leaf relative water content (RWC) was estimated using the formula (Barrs and Weatherley, 1962):

$$\text{Relative Water Content}=\frac{Wf-Wd}{Wt-Wd}\times 100.$$

Wf=fresh weight,Wt=turgid weight,Wd=dry weight

To assess cell membrane stability, the protocol described by Alghabari et al. (2021) was followed. Leaf pieces (100 mg) were placed in two separate tubes, each having deionized water of 20 mL. To measure conductivity, the incubation of a tube was at 40°C for 30 min, while the other tube was kept at 100°C for 10 min. The conductivity of the tubes was tagged C1 and C2 respectively. The formula below was used to estimate the percentage difference:

$$\left\{1-\left(\frac{C1}{C2}\right)*100\right\}$$

#### 2.2.3 Molecular qualities

The RNA from selected plant samples was extracted using the Qiagen RNeasy kit following the protocol established by Li et al. (2018). A cDNA library was generated afterwards according to the same procedure, with 2 µg of RNA used as per the manufacturer’s guidelines. For quantitative real-time polymerase chain reaction (qRT-PCR) analysis, the SYBR Green 1 master kit was employed according to the manufacturer’s instructions. The qRT-PCR thermal cycling conditions involved a primary denaturation step for 10 min at 95°C. Followed by 40 cycles, at 95°C of denaturation for 15 s with a 60°C of annealing/extension for 1 min. During the amplification cycles, fluorescence data was collected to monitor target gene amplification. Gene expression was normalized using the Actin-expressing gene (Vradi03g00210) as a reference, ensuring accurate quantification of gene expression levels in the plant samples. The precise primers employed are detailed in Table 1.

**TABLE 1:** The list of primers used for qRT-PCR analysis.

Primers	Forward	Reverse
SbAPX2	AGT​CGT​GGC​AGT​TGA​GGT​AA	ATC​CTT​GTG​GCA​TCT​TCC​CA
SbCAT3	GGT​TCG​CCG​TCA​AGT​TCT​AC	AAG​AAG​GTG​TGG​AGG​CTC​TC
SbSOD1	ACA​CGG​GAG​AGA​GAG​AGA​GT	TCC​AGC​TCC​AAG​TTT​GCC​T
SbHKT1; 4	ATCGCCATCTGCATCACC	GCC​TCC​CAA​AGA​ACA​TCA​CA
SbDREB2A	AGG​GAC​GAC​AGA​GCA​TAG​G	TGG​CCA​GCA​TCT​GAG​TCT​TC
SbDHN3	GGC​GGA​AGA​AGG​GCA​TCA​G	GTG​TGT​TCT​TGC​TGC​CCG​TA
SbPRP1	GCA​TGC​AAA​TCC​AAA​GTG​CC	CGG​GAA​TTA​ATG​CCG​TCC​AT
SbACTIN	TCC​TGA​AGC​ATC​TTT​CCC​TCC	ACA​GCC​TGA​TTA​GTT​GGG​GG

### 2.3 Statistical analysis

To analyze statistics, the Statistix 8.1 software was employed to perform analysis of variance (ANOVA) at a 5% significance level. Principal component analysis (PCA) was conducted for easier interpretation and visualization of underlying patterns, while correlation and heatmap analyses highlighted significant associations and trends in the data, utilizing RStudio version 1.3.959 (RStudio Team 2020) along with the FactoMineR, corrplot, and pheatmap packages.

## 3 Results

### 3.1 Biochemical qualities

**FIGURE 1:**
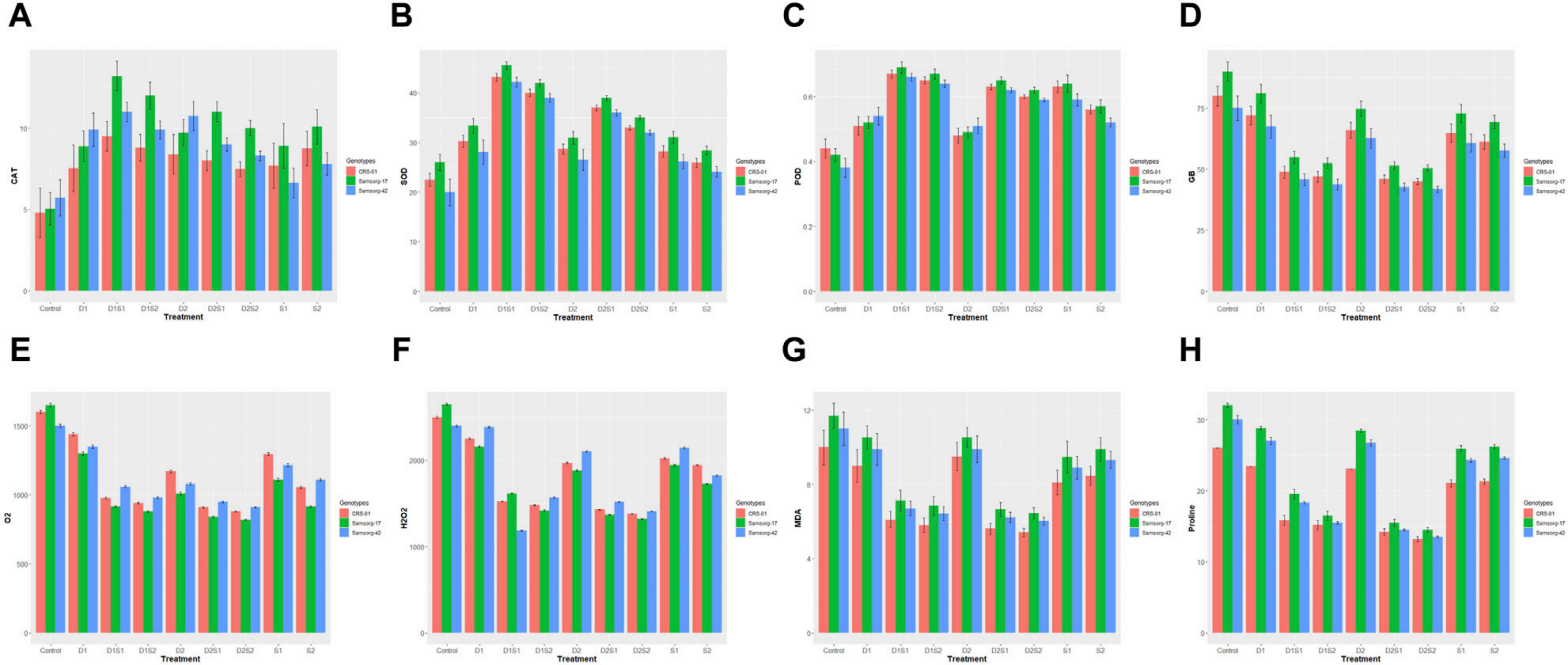
**(A)** Effect of drought and salinity (individual and combined treatments) on catalase (CAT) activities of sorghum genotypes. The vertical bars represent means, and the error bars indicate statistical significance at *p* ≤ 0.05. **(B)** Effect of drought and salinity (individual and combined treatments) on Superoxide dismutase (SOD) activities of sorghum genotypes. The vertical bars represent means, and the error bars indicate statistical significance at *p* ≤ 0.05. **(C)** Effect of drought and salinity (individual and combined treatments) on Peroxidase (POD) activities of sorghum genotypes. The vertical bars represent means, and the error bars indicate statistical significance at *p* ≤ 0.05. **(D)** Effect of drought and salinity (individual and combined treatments) on Glycine betaine (GB) of sorghum genotypes. The vertical bars represent means, and the error bars indicate statistical significance at *p* ≤ 0.05. **(E)** Effect of drought and salinity (individual and combined treatments) on Superoxide anion radical content (O_2_^−^) of sorghum genotypes. The vertical bars represent means, and the error bars indicate statistical significance at *p* ≤ 0.05. **(F)** Effect of drought and salinity (individual and combined treatments) on hydrogen peroxide content (H_2_O_2_) of sorghum genotypes. The vertical bars represent means, and the error bars indicate statistical significance at *p* ≤ 0.05. **(G)** Effect of drought and salinity (individual and combined treatments) on Malondialdehyde (MDA) of sorghum genotypes. The vertical bars represent means, and the error bars indicate statistical significance at *p* ≤ 0.05. **(H)** Effect of drought and salinity (individual and combined treatments) on proline content of sorghum genotypes. The vertical bars represent means, and the error bars indicate statistical significance at *p* ≤ 0.05.

Under control conditions, all three genotypes had relatively low CAT activity (Figure 1A). Apart from control and the single drought treatments (D1 and D2) where Samsorg-42 showed the highest CAT activity, Samsorg-17 consistently exhibits the highest CAT activity across all stress conditions. Under salinity stress (S1 and S2), CAT activity increases in all genotypes but to a lesser extent compared to drought stress or combined stress conditions. CAT activity is generally higher in the combined stress treatments compared to individual stress conditions.

Under control conditions, all three genotypes exhibited relatively low SOD activity, with Samsorg-17 showing the highest activity (Figure 1B). This trend was maintained in all stress conditions. Combined stress conditions, especially D1S1, generally induced higher SOD activity than single stress treatments. The SOD activity in the single stress treatment was similar.

The control group exhibited baseline POD levels, setting the stage for assessing stress-induced deviations (Figure 1C). CRS-01 had the most activity of POD under this no stress condition. Under drought conditions (D1 and D2), a marked increase in POD activity was observed, with genotype Samsorg-42 showing the most pronounced response. Isolated salinity treatments (S1, S2) and combined treatments painted a different picture with Samsorg-17 showing the most POD activity.

Under control and all stress conditions, Samsorg-17 showed the highest GB levels (Figure 1D). Overall, the treatments had GB levels lower to the control. Single treatments of drought and salinity resulted in higher GB levels, compared to the concurrent treatments.

The control with Samsorg-17 displayed the highest overall O₂⁻, H₂O₂, MDA and proline levels (Figures 1E–H). O₂⁻, H₂O₂, MDA and proline levels were greater in the separate treatments of drought and salinity compared to the combined treatments. CRS-01 exhibited a more notable increase in these treatments except for S2.

In S2 and the combined treatments, a more significant increase in O₂⁻was observed in Samsorg-42. Samsorg-42 also recorded the highest H₂O₂ levels in all treatments except for D1S1 where Samsorg-17 had the highest. Samsorg-17 also had the most elevation in MDA and proline levels in all treatments and control (Figure 1).

### 3.2 Physiological measurements

**FIGURE 2:**
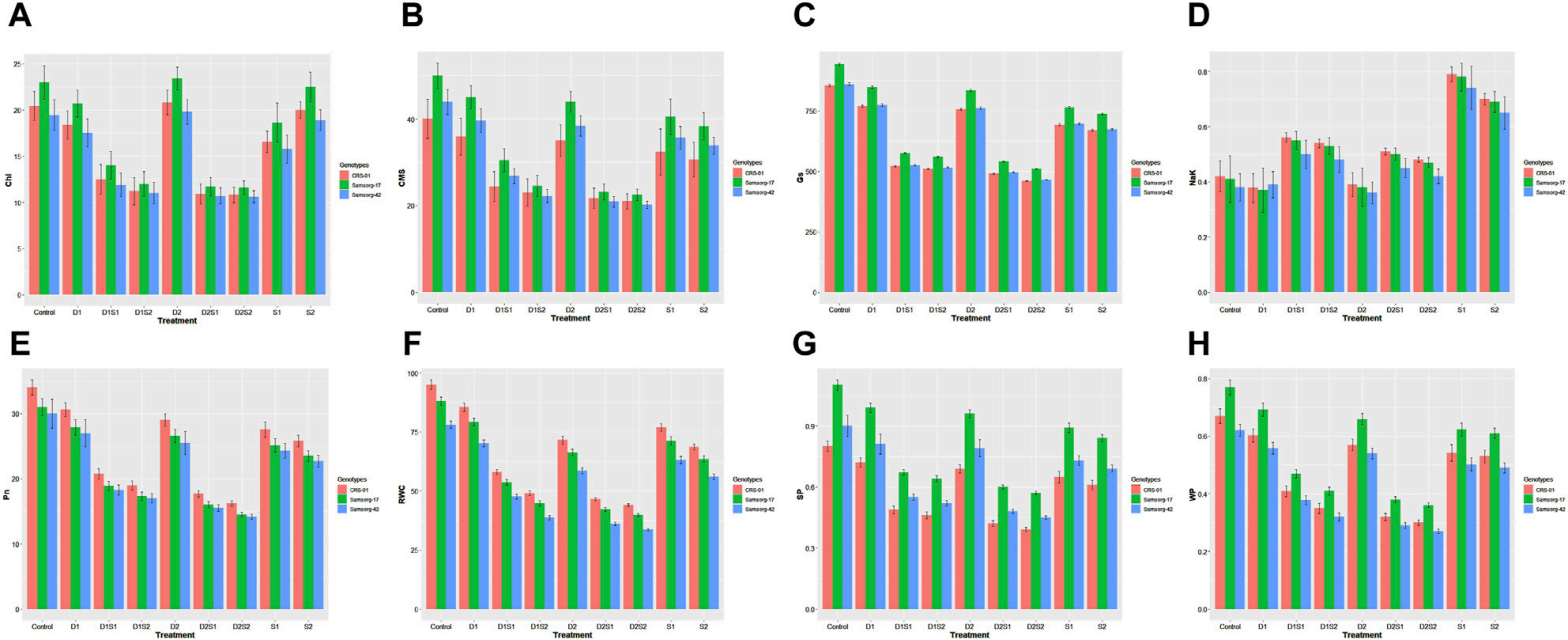
**(A)** Effect of drought and salinity (individual and combined treatments) on Chlorophyll content (Chl) of sorghum genotypes. The vertical bars represent means, and the error bars indicate statistical significance at *p* ≤ 0.05. **(B)** Effect of drought and salinity (individual and combined treatments) on Cell membrance stability (CMS) of sorghum genotypes. The vertical bars represent means, and the error bars indicate statistical significance at *p* ≤ 0.05. **(C)** Effect of drought and salinity (individual and combined treatments) on Stomatal conductance (Gs) of sorghum genotypes. The vertical bars represent means, and the error bars indicate statistical significance at *p* ≤ 0.05. **(D)** Effect of drought and salinity (individual and combined treatments) on Na/K ratio of sorghum genotypes. The vertical bars represent means, and the error bars indicate statistical significance at *p* ≤ 0.05. **(E)** Effect of drought and salinity (individual and combined treatments) on Photosynthetic rate (Pn) of sorghum genotypes. The vertical bars represent means, and the error bars indicate statistical significance at *p* ≤ 0.05. **(F)** Effect of drought and salinity (individual and combined treatments) on Relative water content (RWC) of sorghum genotypes. The vertical bars represent means, and the error bars indicate statistical significance at *p* ≤ 0.05. **(G)** Effect of drought and salinity (individual and combined treatments) on Solute potential (SP) of sorghum genotypes. The vertical bars represent means, and the error bars indicate statistical significance at *p* ≤ 0.05. **(H)** Effect of drought and salinity (individual and combined treatments) on Water potential (WP) of sorghum genotypes. The vertical bars represent means, and the error bars indicate statistical significance at *p* ≤ 0.05.

Under control conditions, all genotypes showed higher chlorophyll content, compared with most treatments (Figure 2A). However, overall, most pronounced induction of chlorophyll content was recorded at the higher drought level (D2). While the chlorophyll contents in the individual treatments of drought and salinity were comparable, a significant reduction was noticed in the combination treatments. Samsorg-17 revealed the highest chlorophyll contents under all treatments.

Furthermore, all genotypes exhibit robust CMS levels under control conditions (Figure 2B). As the separate drought and salinity stress intensifies, CMS diminish across genotypes, further CMS decline was experienced under simultaneous stresses of drought and salinity. Similar trend with CMS was also noticed in the stomatal conductance (Gs) (Figure 2C). Samsorg-17 also displayed highest CMS and stomatal conductance in the control and all stress conditions.

The graph showcases the control conditions of Na/K ratios, with all genotypes displaying similar ratios (Figure 2D). As the drought progresses, a less discernible reduction in the Na/K ratio is observed across genotypes. Interestingly, when salinity is introduced alongside drought in the combined treatments, the Na/K ratio slightly increased compared to drought alone. Under isolated salinity conditions, the genotypes exhibit significant elevation in the Na/K ratio. CRS-01 depicted the overall highest Na/K ratio in the treatments and control. CRS-01, followed by Samsorg-17, equally displayed most Pn and RWC levels right from the control and throughout all the treatments (Figures 2E, F). Compared with control, drought and salinity conditions led to a decrease in Pn and RWC for all genotypes, with a more pronounced reduction at the integrated treatments.

Drought and salt treatments lead to a decrease in SP and WP as compared to the control (Figures 2G, H). However, the combined drought and salinity treatment resulted in the lowest sp and wp values, highlighting the compounded stress effect. Overall, while Samsorg-17 followed by Samsorg-42 had highest sp, Samsorg-17 followed by CRS-01 had the highest wp.

### 3.3 Gene expression analysis

**FIGURE 3:**
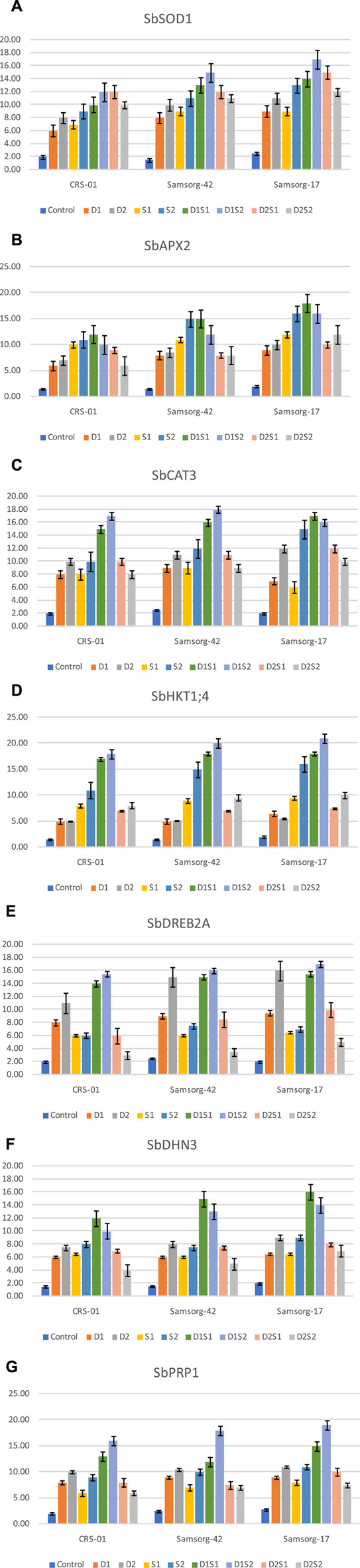
**(A)** Relative expression of SbSOD1 gene in various sorghum genotypes under both individual and combined stresses of drought and salinity. **(B)** Relative expression of SbAPX2 gene in various sorghum genotypes under both individual and combined stresses of drought and salinity. **(C)** Relative expression of SbCAT3 gene in various sorghum genotypes under both individual and combined stresses of drought and salinity. **(C)** Relative expression of SbHKT1; 4 gene in various sorghum genotypes under both individual and combined stresses of drought and salinity. **(E)** Relative expression of SbDREB2A gene in various sorghum genotypes under both individual and combined stresses of drought and salinity. **(F)** Relative expression of SbDHN3 gene in various sorghum genotypes under both individual and combined stresses of drought and salinity. **(G)** Relative expression of SbPRP1 gene in various sorghum genotypes under both individual and combined stresses of drought and salinity.

Outcomes of gene expression are illustrated in (Figure 3). Expression analysis of SbSOD1 revealed an upregulation under both drought and salinity across all genotypes. The genotypes however exhibited the highest expression levels under the combined stress condition (especially D1S2). Overall, Samsorg-17 displayed the most significant increase across the treatments. SbAPX2 expression followed a similar trend to SbSOD1 under stress conditions. The peak expression for the genotypes was also under D1S1. Also, Samsorg-17 demonstrated the most pronounced expression over all the treatments. For SbCAT3, drought conditions alone (D1 and D2) and severe salinity (S2) significantly elevated expression levels, but the highest expression overall was recorded under combined treatments involving moderate drought (D1S1, D1S2) across all the genotypes. SbHKT1; 4 expression was notably enhanced under salinity (especially S2) compared to drought stress. The most substantial increase was however seen under combined stress (particularly D1S2) in all genotypes. Expression levels of SbDREB2A were significantly upregulated under drought and combined stress conditions, peaking at D1S2 across CRS-01, Samsorg-42, and Samsorg-17. SbDHN3 expression increased markedly under the stress conditions, with the highest expression under D1S1 in all genotypes. SbPRP1 exhibited increased expression under stress conditions particularly drought, severe salinity (S2) and combined stress conditions, with the highest levels recorded under D1S2 for all genotypes.

### 3.4 Correlation analysis

**FIGURE 4:**
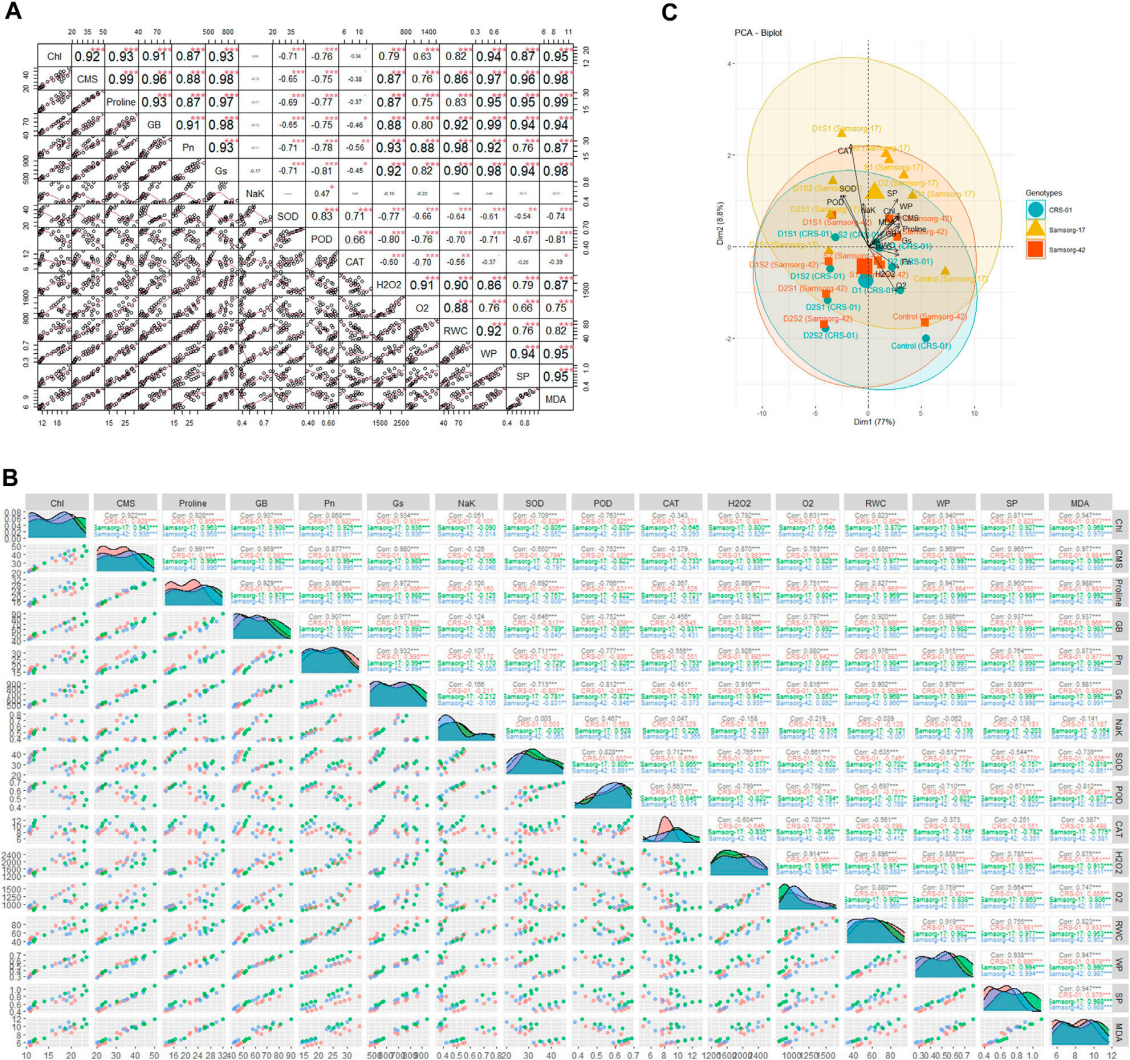
**(A)** The correlations among factors. The upper matrix shows the Pearson coefficients, and results were significant at ***p < 0.01, **p < 0.05, or *p < 0.1 as marked. The red solid lines in the lower matrix show a smooth regression between the two factors. **(B)** Pearson correlation matrices for the effects of individual and combined drought and salinity stresses on sorghum genotypes, with significance levels denoted as follows: * (*p* ≤ 0.1), ∗∗ (*p* ≤ 0.01), and ∗∗∗ (*p* ≤ 0.001). **(C)** PCA scatter plot showing the grouping of physiological and biochemical characteristics according to their resemblance and variation, particularly concerning various sorghum genotypes.

The PCA biplot analysis of sorghum genotypes under varying drought and salinity stress conditions reveals significant insights into the association and divergence of physiological, biochemical, and growth-related traits (Figure 4C). Traits such as catalase (CAT), superoxide dismutase (SOD), and peroxidase (POD) activities, along with solute potential (SP), water potential (WP), and relative water content (RWC), appear to be closely associated, as indicated by the vectors pointing in similar directions. Genotypes showed a clustered response under control conditions, suggesting a similar baseline for physiological and biochemical traits. Under stress conditions, genotypes that maintain higher RWC, WP, and lower SP tend to cluster together, indicating a shared mechanism of stress tolerance. The spread of genotypes across the biplot under D and S conditions indicates a divergence in how each genotype copes with stress, with some maintaining better physiological balance than others.

The correlation matrix highlights how different physiological and biochemical traits are interrelated in contributing to the stress tolerance of sorghum genotypes (Figures 4A, B). For instance, Chl, CMS, Proline, and RWC exhibit strong positive correlations across all genotypes and treatments. Pn, Gs, SOD, and WP show moderate positive correlations with several parameters. NaK, POD, CAT, H₂O₂, O₂⁻, and MDA generally show low or negative correlations with other parameters.

### 3.5 Heatmap analysis

**FIGURE 5:**
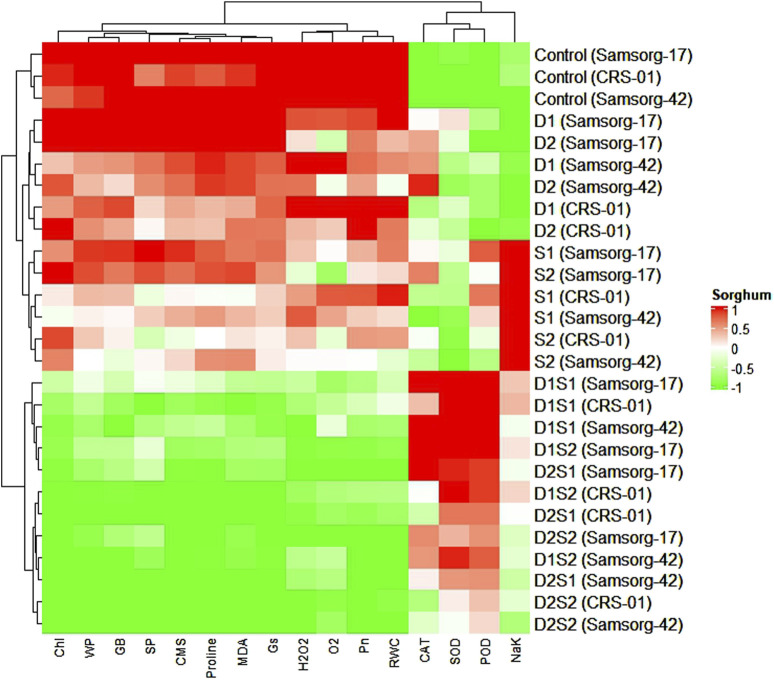
Cluster dendrogram heatmap depicting how physiological and biochemical traits respond in sorghum genotypes under stress conditions of individual and combined drought and salinity.

The heatmap provides a comprehensive overview of the biochemical and physiological responses of three sorghum genotypes under different treatment conditions (Figure 5). The hierarchical clustering of traits reveals groups of positively or negatively correlated traits. For example, traits such as CMS, proline, and MDA cluster together. The control samples for each genotype generally show a distinct profile (green) compared to stressed samples (red). Under drought stress (D1, D2), genotypes exhibit increased levels of proline and MDA.

Salinity stress (S1, S2) leads to increased NaK ratio and MDA, with a notable reduction in traits like RWC and WP. Combined drought and salinity stress (D1S1, D2S1, D1S2, D2S2) show an intensified stress response with high proline and MDA levels, and reduced chlorophyll content and photosynthetic rate.

## 4 Discussion

The integrated stress responses in sorghum, particularly under the combined influence of drought and salinity, reveal complex interactions between biological and physiological parameters, as well as gene expression patterns.

The assessment of enzymatic activities such as superoxide dismutase (SOD), peroxidase (POD), and catalase (CAT) sheds light on the antioxidant defense mechanisms in sorghum plants (Figures 1A–C). The observed variations in enzyme units among treatments and genotypes highlight the genotype-specific responses to stress. For instance, genotype CRS-01 exhibits lower SOD levels but higher CAT levels compared to other genotypes under stress conditions, indicating a differential regulation of antioxidant enzymes (Vela-Hinojosa et al., 2019). Moreover, the gradual escalation of drought stress leads to a step-wise increase in proline levels across genotypes, indicating its role as a responsive osmo-protectant (Zhang et al., 2022). Similarly, glycine betaine accumulation is influenced by both genotype and stress (Figure 1D), with CRS-01 and Samsorg-42 demonstrating proficiency in accumulating this osmolyte, suggesting potential adaptive strategies for osmotic adjustment (Annunziata et al., 2019).

Physiological parameters further elucidate the response mechanisms of sorghum genotypes to stress (Figures 2A–H). Chlorophyll content, Na/K ratio, Relative Water Content (RWC), and water potential serve as indicators of stress tolerance (Ramani et al., 2023). Genotype CRS-01, particularly under treatment D1S2, exhibits significant effects on chlorophyll content and water potential, implying its resilience to combined drought and salinity stresses.

Additionally, the decrease in photosynthesis rate (Pn) and stomatal conductance (Gs) with increasing stress severity underscores the trade-off between water conservation and photosynthetic activity under stress conditions (Zou et al., 2022). Despite this general trend, Samsorg-17 consistently displays higher Pn and Gs values across treatments, indicating its inherent resilience to stress.

The gene expression analysis provides molecular insights into the adaptive responses of sorghum genotypes to stress. Upregulation of genes involved in antioxidant defense (SbSOD1, SbAPX2, SbCAT3), ion homeostasis (SbHKT1; 4), and stress tolerance (SbDREB2A, SbDHN3, SbPRP1) reflects the activation of stress-responsive pathways (Figure 3) (Ma et al., 2022). Overall, the expression levels of the studied genes (SbSOD1, SbAPX2, SbCAT3, SbHKT1; 4, SbDREB2A, SbDHN3, and SbPRP1) consistently increased under stress conditions compared to control. Aside, SbHKT1; 4 and SbAPX2, drought show higher expression than salinity in the genes. Generally, the combined stress conditions, particularly D1S2, often resulted in the highest expression levels establishing the severity of the integrated stresses on sorghum growth (Angon et al., 2022).

The correlation analysis elucidates the intricate relationships between physiological, and biochemical traits in sorghum genotypes subjected to drought and salinity stresses (Figures 4A, B). The PCA biplot underscores the clustering of catalase (CAT), superoxide dismutase (SOD), and peroxidase (POD) activities, along with solute potential (SP), water potential (WP), and relative water content (RWC) (Figure 4C). This clustering implies a concerted response among these traits in mitigating oxidative stress and maintaining cellular homeostasis under adverse conditions (Mirzamasoumzadeh and Mollasadeghi, 2019; Song et al., 2020). The tight clustering of genotypes under control conditions suggests a uniform baseline in physiological and biochemical traits, reflective of their inherent genetic makeup.

In contrast, the dispersion of genotypes under stress conditions highlights the differential adaptive mechanisms employed by each genotype (Figures 4A, B) (Abdel-Ghany et al., 2020). Genotypes, like Samsorg-17, that maintained higher RWC and WP, alongside lower SP, tend to cluster, indicating a shared mechanism of stress tolerance, possibly through efficient osmotic adjustment and water retention strategies (Hasanuzzaman et al., 2023). These genotypes exhibit a robust physiological balance, conferring resilience against drought and salinity stresses.

The correlation matrix further delineates the interdependencies among various traits. Strong positive correlations among chlorophyll content (Chl), cell membrane stability (CMS), proline, and RWC across all genotypes and treatments suggest these traits’ pivotal roles in enhancing stress tolerance (Nowsherwan et al., 2018; Falaknaz et al., 2019). Proline accumulation, a known osmo-protectant, likely contributes to maintaining RWC and stabilizing cellular structures, as indicated by its strong positive correlation with CMS and RWC (Falaknaz et al., 2019; Alsamadany et al., 2024). Moderate positive correlations of photosynthetic rate (Pn), stomatal conductance (Gs), SOD, and WP with several parameters highlight their contributions to sustaining photosynthetic efficiency and antioxidative defense under stress. Conversely, traits such as Na/K ratio, POD, CAT, hydrogen peroxide (H_2_O_2_), superoxide anion (O₂⁻), and malondialdehyde (MDA) exhibit low or negative correlations with other parameters, suggesting their variable roles in stress responses, possibly linked to specific stress conditions or thresholds (Chen et al., 2023).

The heatmap analysis provides a holistic view of the physiological and biochemical responses of sorghum genotypes under varying stress conditions, revealing distinct patterns of trait clustering (Figure 5). The hierarchical clustering indicates that traits such as CMS, proline, and MDA are closely associated, reflecting their collective involvement in stress responses (Lian et al., 2021).

The distinct profile of control samples (green) compared to stressed samples (red) highlights the significant impact of both drought and salinity stresses on the biochemical and physiological status of the genotypes (Eisenring et al., 2023). Under drought stress (D1, D2), the increased levels of proline and MDA indicate heightened osmo-protective and antioxidative responses. Proline accumulation aids in osmotic adjustment, while increased MDA levels suggest lipid peroxidation, a marker of oxidative stress (Abdulbaki et al., 2024). Salinity stress (S1, S2) exacerbates ionic imbalances, as evidenced by the increased Na/K ratio and MDA levels, coupled with reductions in RWC and WP. This indicates that salinity stress imposes additional ionic and osmotic challenges, impairing water uptake and cellular integrity (Joshi et al., 2022).

As established by Angon et al. (2022), combined drought and salinity stress (D1S1, D2S1, D1S2, D2S2) elicits a heightened stress response, marked by increased proline and MDA levels, alongside decreased chlorophyll content and photosynthetic rates. The simultaneous rise in proline and MDA indicates a synergistic effect, prompting genotypes to activate osmo-protective and antioxidative mechanisms to mitigate the compounded stress (Abdulbaki et al., 2024). The decline in chlorophyll and photosynthetic rates highlights the significant impact on the photosynthetic apparatus, likely due to increased oxidative damage and stomatal limitations.

Furthermore, under combined stress conditions, the upregulation of antioxidant defense genes (SbSOD1, SbAPX2, SbCAT3) was more significant than under individual stress treatments. This increased gene expression is linked to enhanced activities of antioxidant enzymes, essential for scavenging reactive oxygen species (ROS) and protecting cells during stress. In agreement with this finding, Wang et al. (2021) reported the upregulation of antioxidant gene expression in response to oxidative stress. The highest expression levels of these genes in Samsorg-17, along with elevated proline and glycine betaine levels, suggest a coordinated molecular response that boosts its resilience to combined drought and salinity stress. Correspondingly, the improved growth of plant under stress due to the sequence combination of antioxidants and proline was also reported by El-Beltagi et al. (2020).

Stress conditions also significantly affected physiological parameters, including chlorophyll content, cell membrane stability (CMS), stomatal conductance (Gs), and water potential (WP). The additional reduction in chlorophyll and photosynthesis rate under combined stress underscores the compounded negative effects on photosynthesis. Similarly, in comparison to individual occurrence of stress, combined stresses lead to a more significant decrease in photosynthetic carbon gain under fluctuating light conditions in tomato (Zeng et al., 2024). However, Samsorg-17 exhibited higher chlorophyll levels and photosynthesis, indicating effective protective mechanisms against oxidative stress, likely due to the upregulation of SbSOD1 and SbCAT3.

In conclusion, the current study investigated the integrated stress responses of *Sorghum bicolor* to combined drought and salinity stresses, alongside individual stress conditions. The key findings highlight significant genotype-specific variations in physiological, biochemical, and molecular responses. Samsorg-17 exhibited the highest resilience, maintaining superior physiological traits such as higher chlorophyll content, cell membrane stability, stomatal conductance, and water potential. This genotype also showed elevated levels of antioxidative enzymes (CAT, SOD, POD) and osmolytes (proline and glycine betaine), which are critical for stress tolerance. The upregulation of stress-responsive genes (SbSOD1, SbAPX2, SbCAT3, SbHKT1; 4, SbDREB2A, SbDHN3, and SbPRP1) was most pronounced under combined drought and salinity conditions. Samsorg-17 displayed the highest expression levels of these genes, correlating with its robust physiological and biochemical performance. Correlation and PCA analyses revealed strong interdependencies among traits related to antioxidative defence, osmotic adjustment, and stress tolerance, indicating a coordinated response to mitigate oxidative stress and maintain cellular homeostasis. Overall, Samsorg-17’s superior stress resilience underscores the importance of enhancing antioxidative defence mechanisms and osmotic adjustment capabilities in sorghum breeding programs.

These observations provide a comprehensive understanding of the adaptive responses in sorghum, contributing to the development of stress-tolerant cultivars for sustainable agriculture amidst climate variability.

## Data Availability

The original contributions presented in the study are included in the article/supplementary material, further inquiries can be directed to the corresponding author.
