# Supplementary figures and images for: Genotypic variability in stress responses of Sorghum bicolor under drought and salinity conditions
Source: Front Genet. 2025 Jan 8;15:1502900. doi: 10.3389/fgene.2024.1502900 (PMC11750996; doi:10.3389/fgene.2024.1502900)

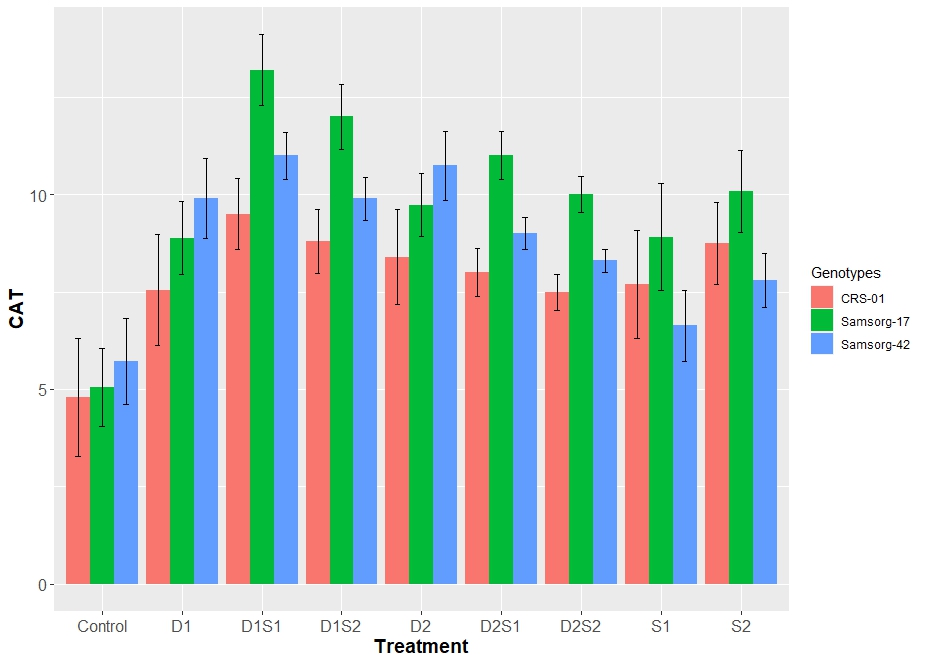

Supplement: Supplementary file 2 [file DataSheet1.zip › 1.jpeg]

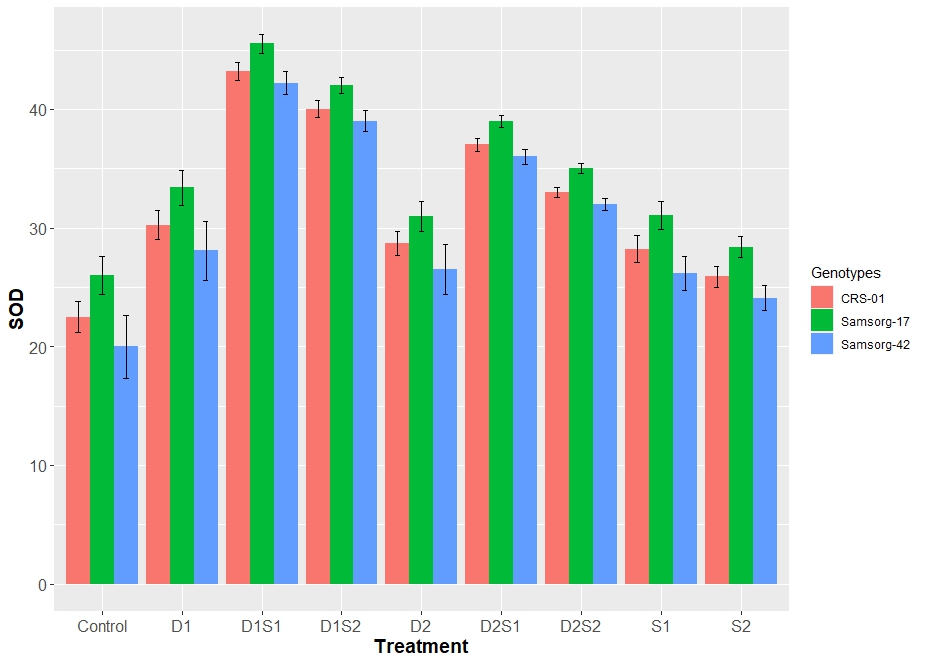

Supplement: Supplementary file 2 [file DataSheet1.zip › 2.jpeg]

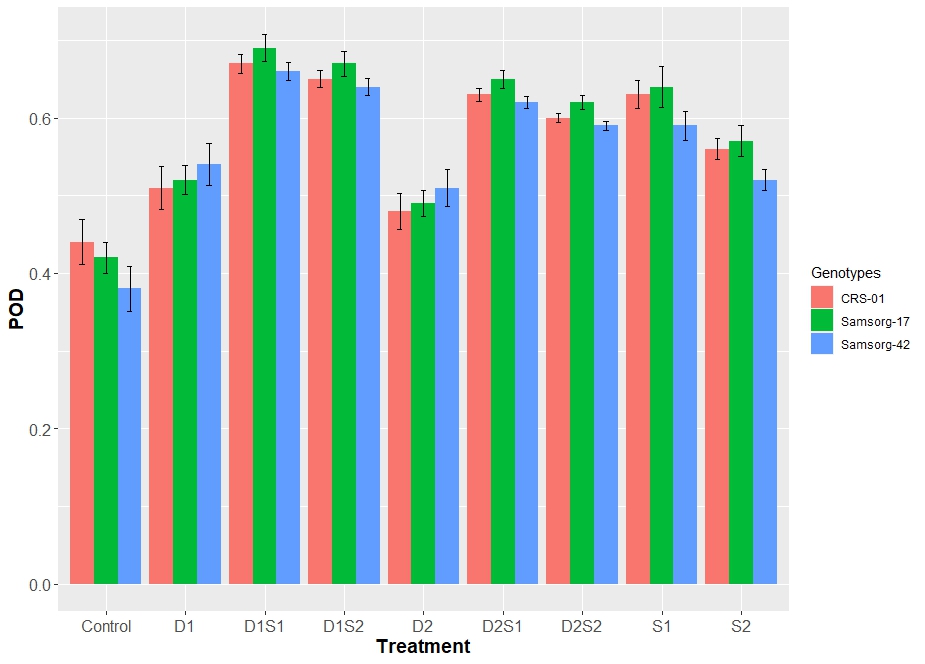

Supplement: Supplementary file 2 [file DataSheet1.zip › 3.jpeg]

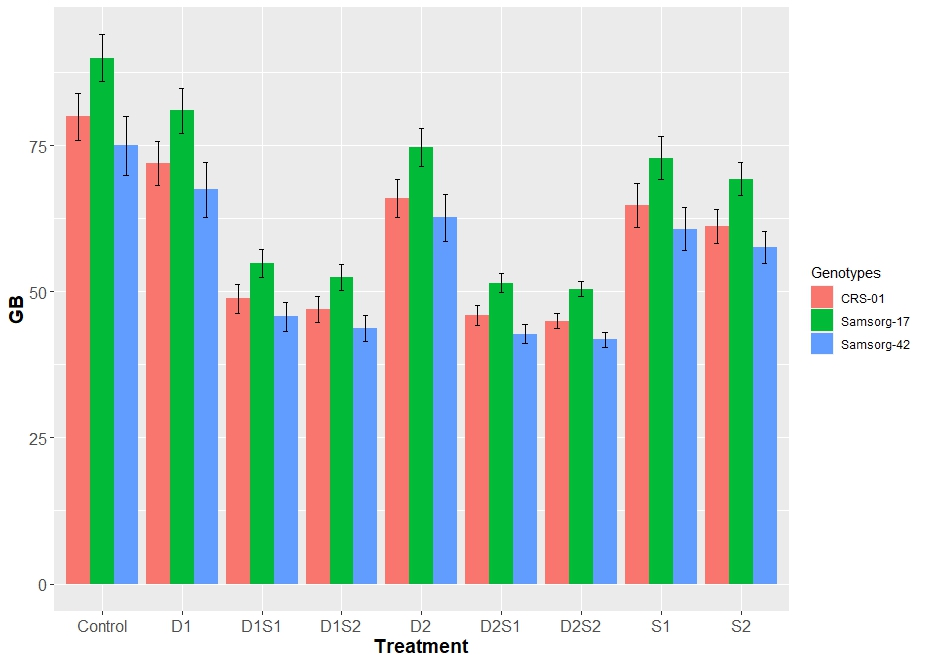

Supplement: Supplementary file 2 [file DataSheet1.zip › 4.jpeg]

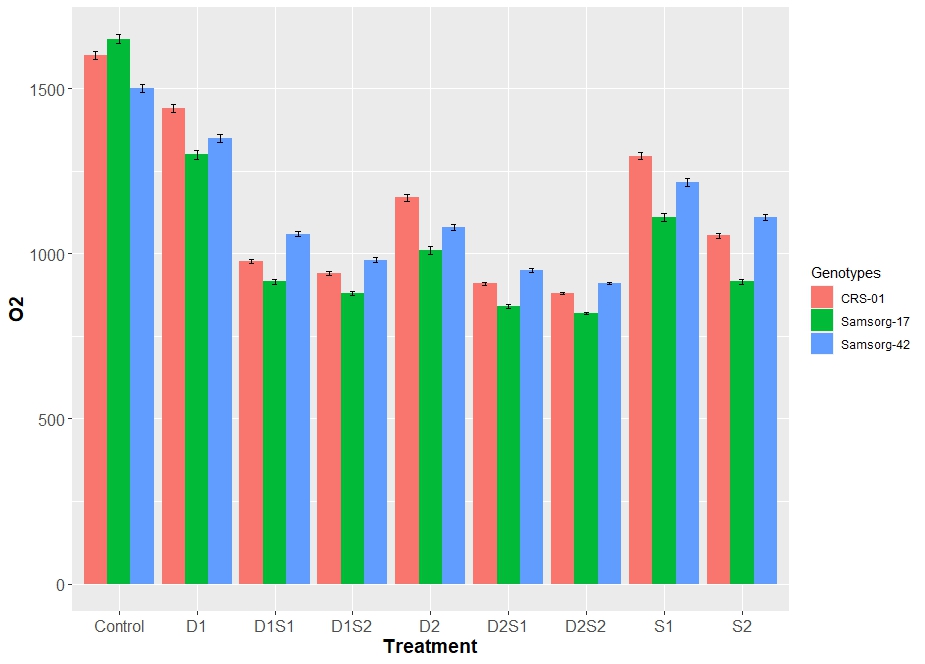

Supplement: Supplementary file 2 [file DataSheet1.zip › 5.jpeg]

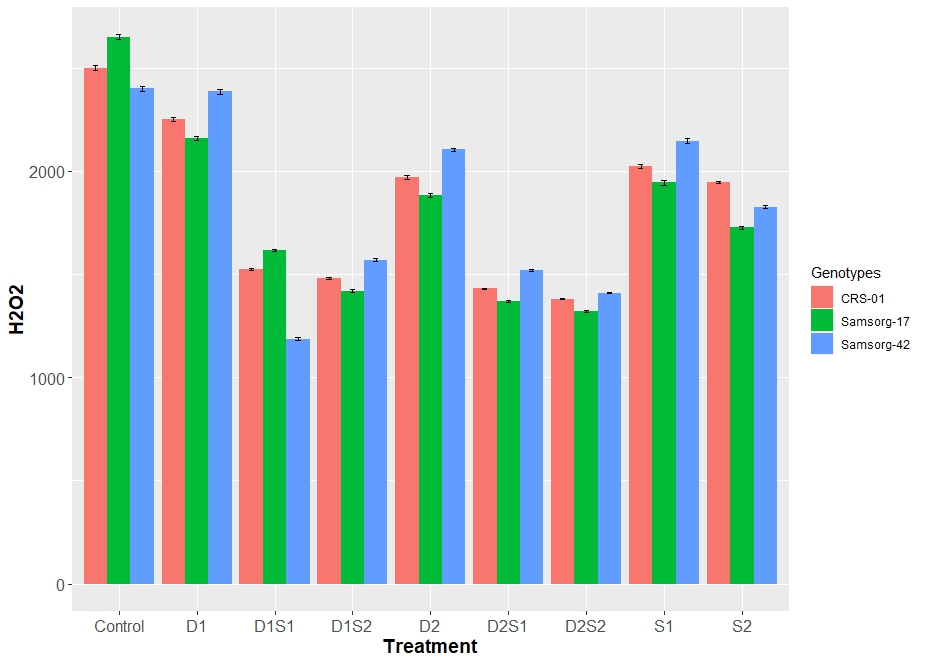

Supplement: Supplementary file 2 [file DataSheet1.zip › 6.jpeg]

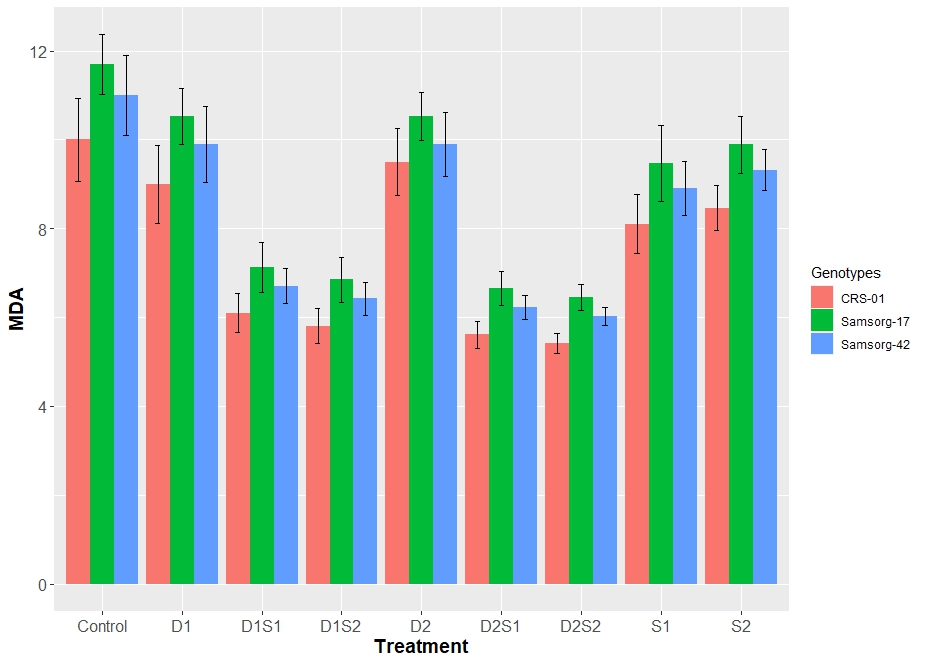

Supplement: Supplementary file 2 [file DataSheet1.zip › 7.jpeg]

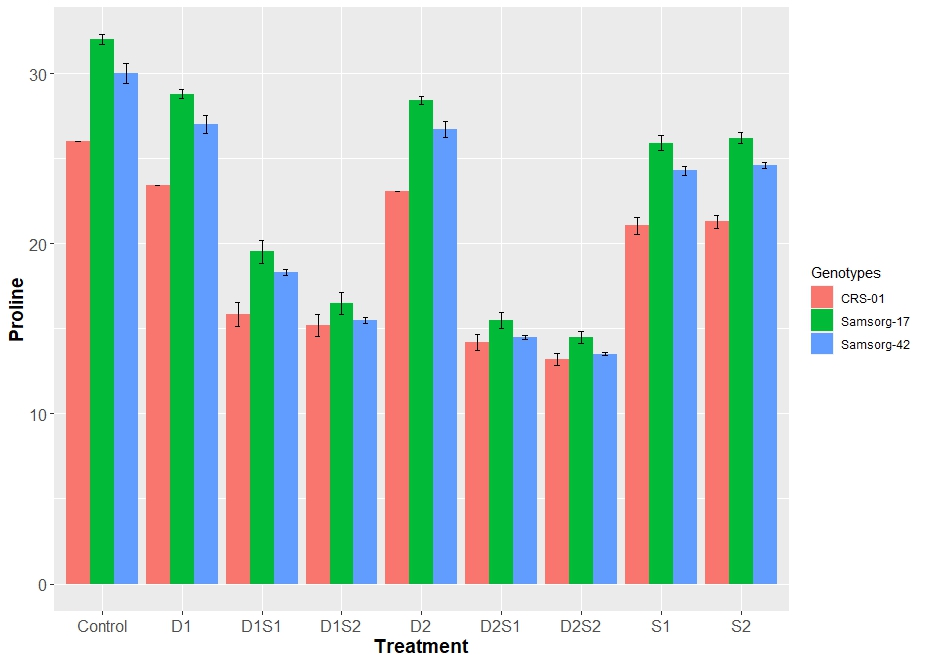

Supplement: Supplementary file 2 [file DataSheet1.zip › 8.jpeg]

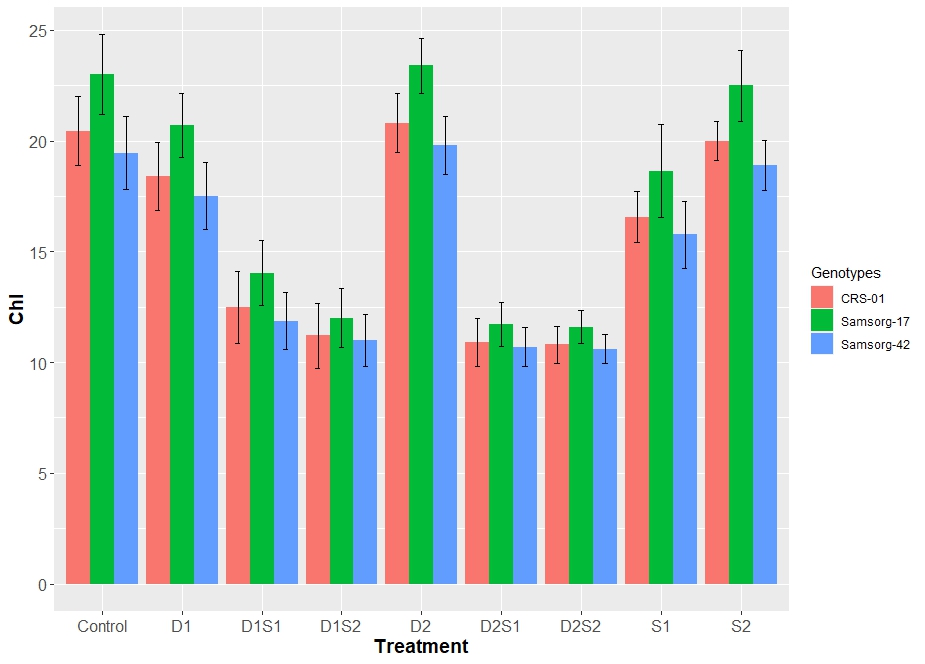

Supplement: Supplementary file 2 [file DataSheet1.zip › 9.jpeg]

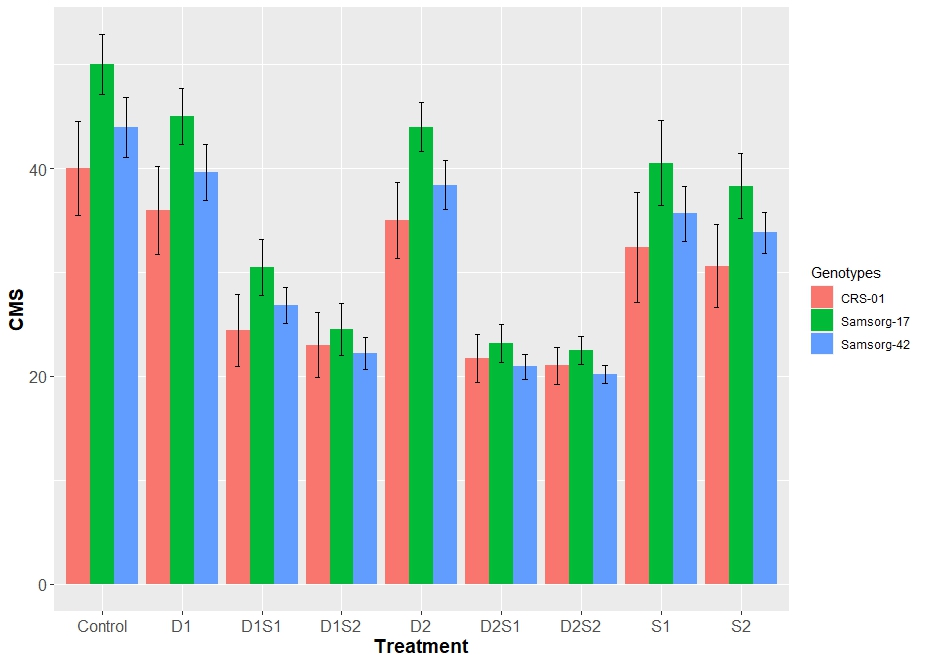

Supplement: Supplementary file 2 [file DataSheet1.zip › 10.jpeg]

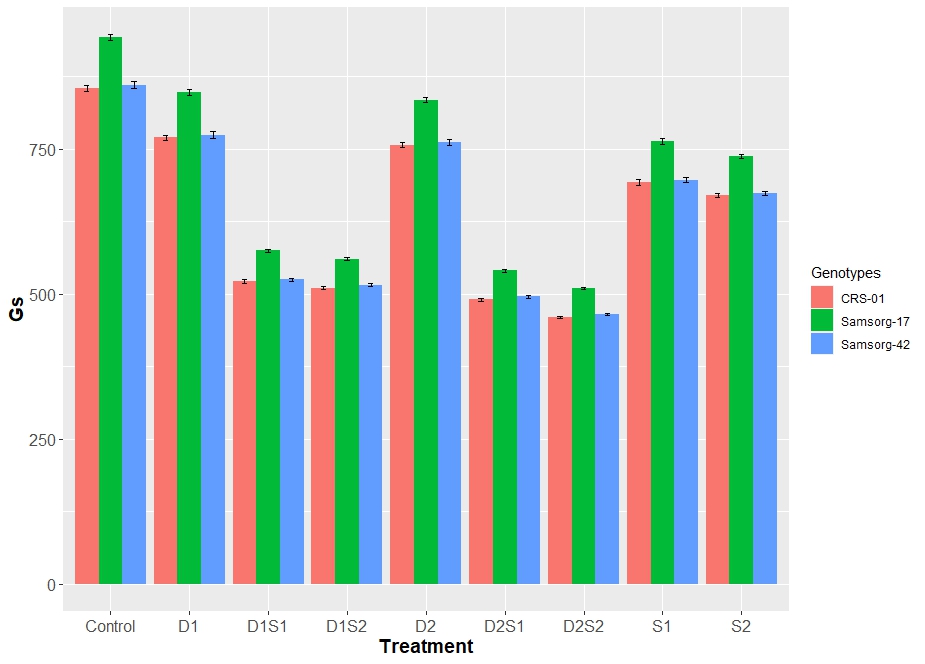

Supplement: Supplementary file 2 [file DataSheet1.zip › 11.jpeg]

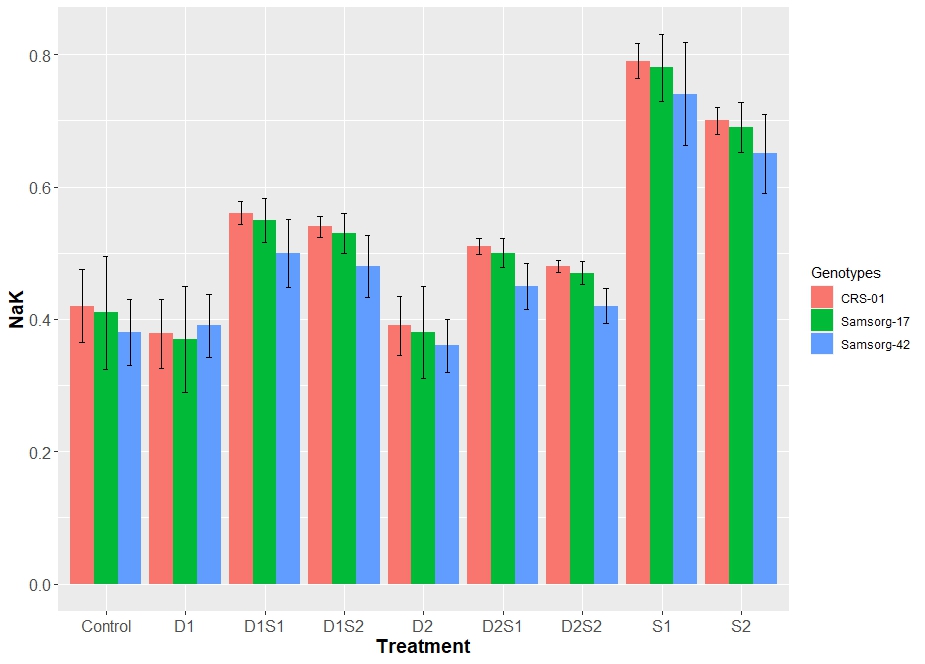

Supplement: Supplementary file 2 [file DataSheet1.zip › 12.jpeg]

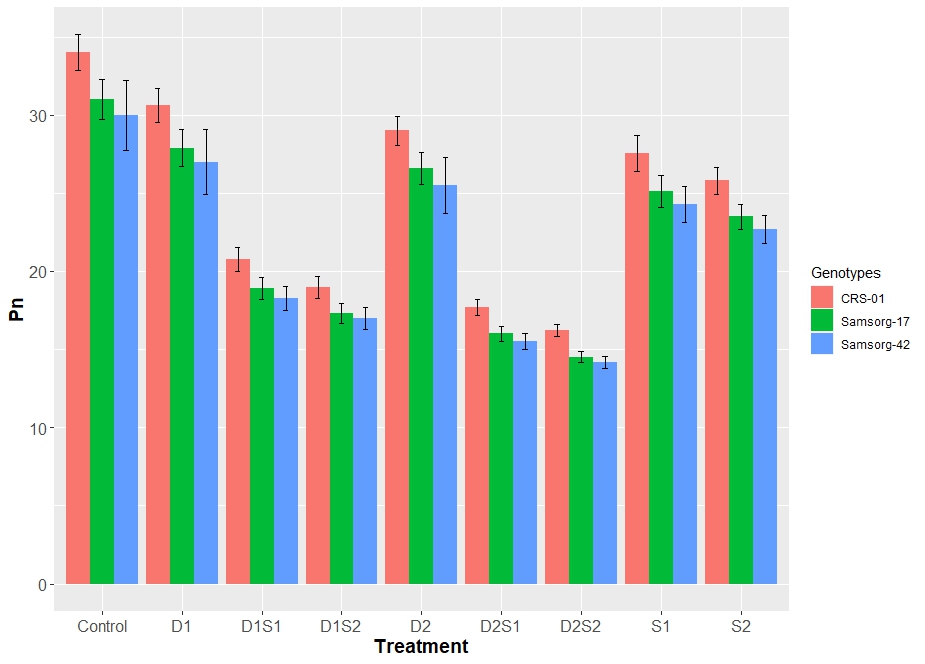

Supplement: Supplementary file 2 [file DataSheet1.zip › 13.jpeg]

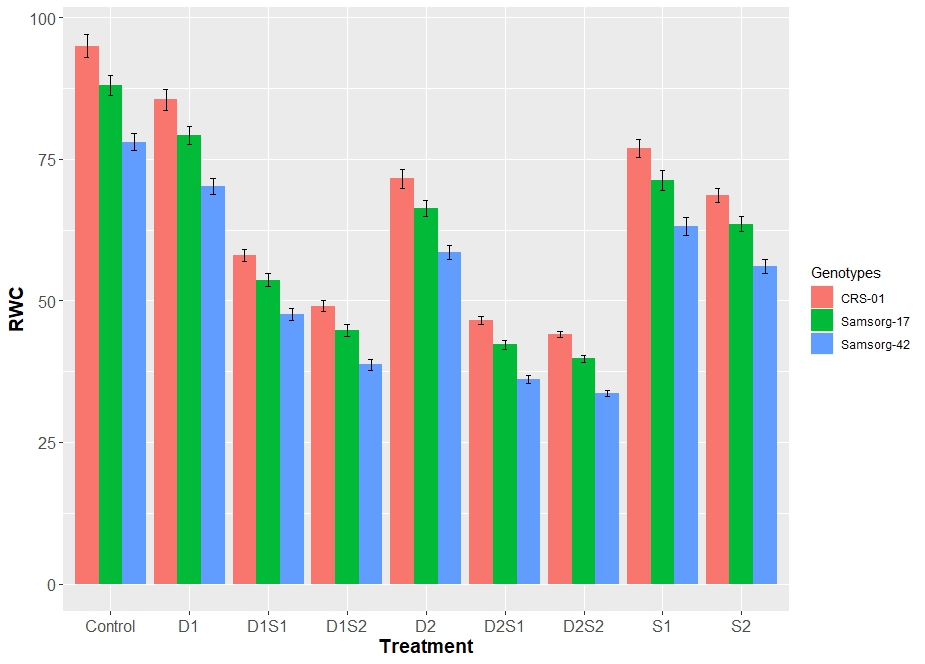

Supplement: Supplementary file 2 [file DataSheet1.zip › 14.jpeg]

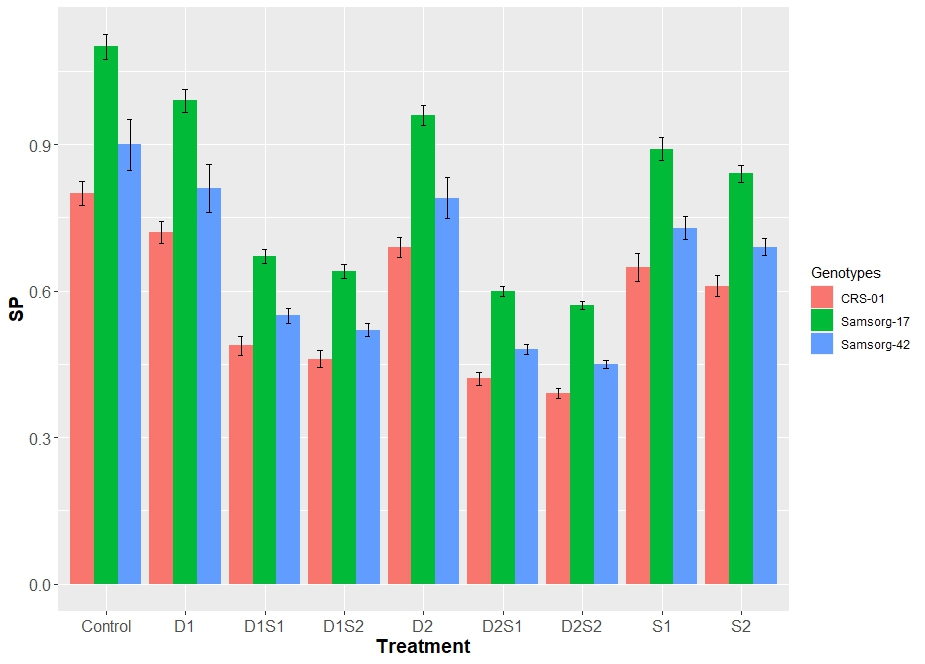

Supplement: Supplementary file 2 [file DataSheet1.zip › 15.jpeg]

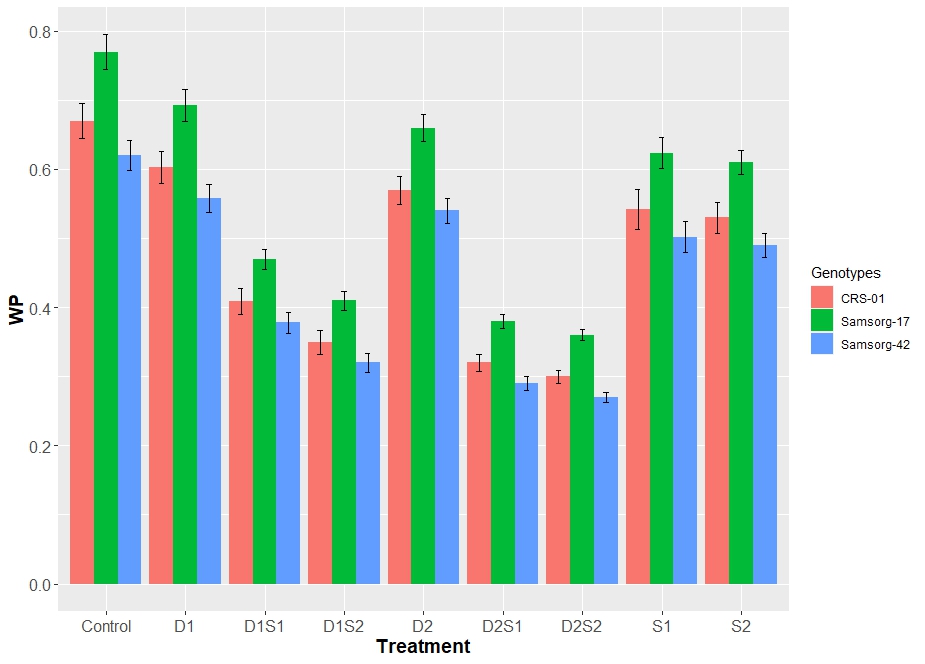

Supplement: Supplementary file 2 [file DataSheet1.zip › 16.jpeg]

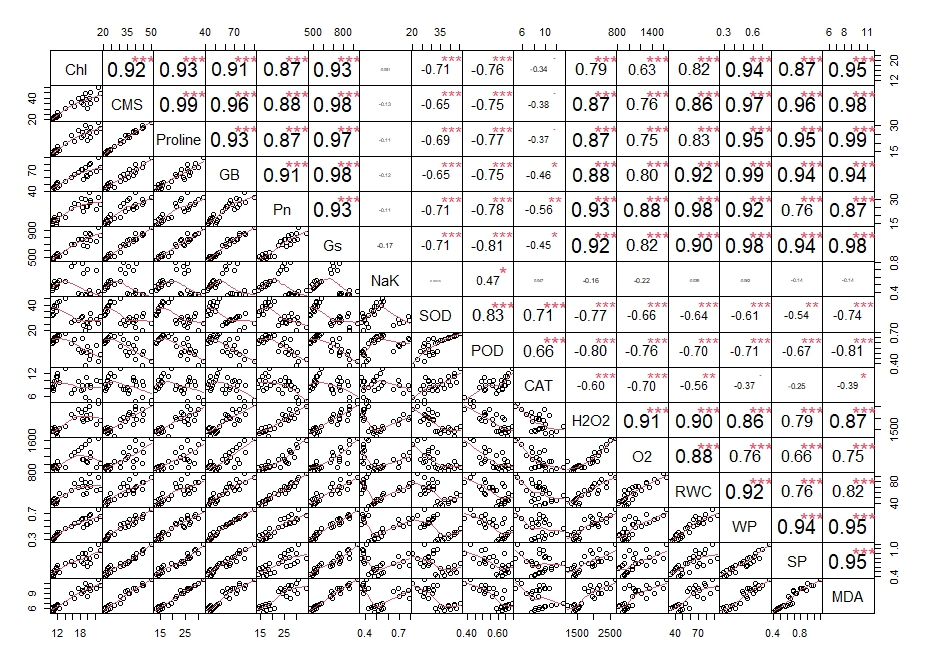

Supplement: Supplementary file 2 [file DataSheet1.zip › 17.jpeg]

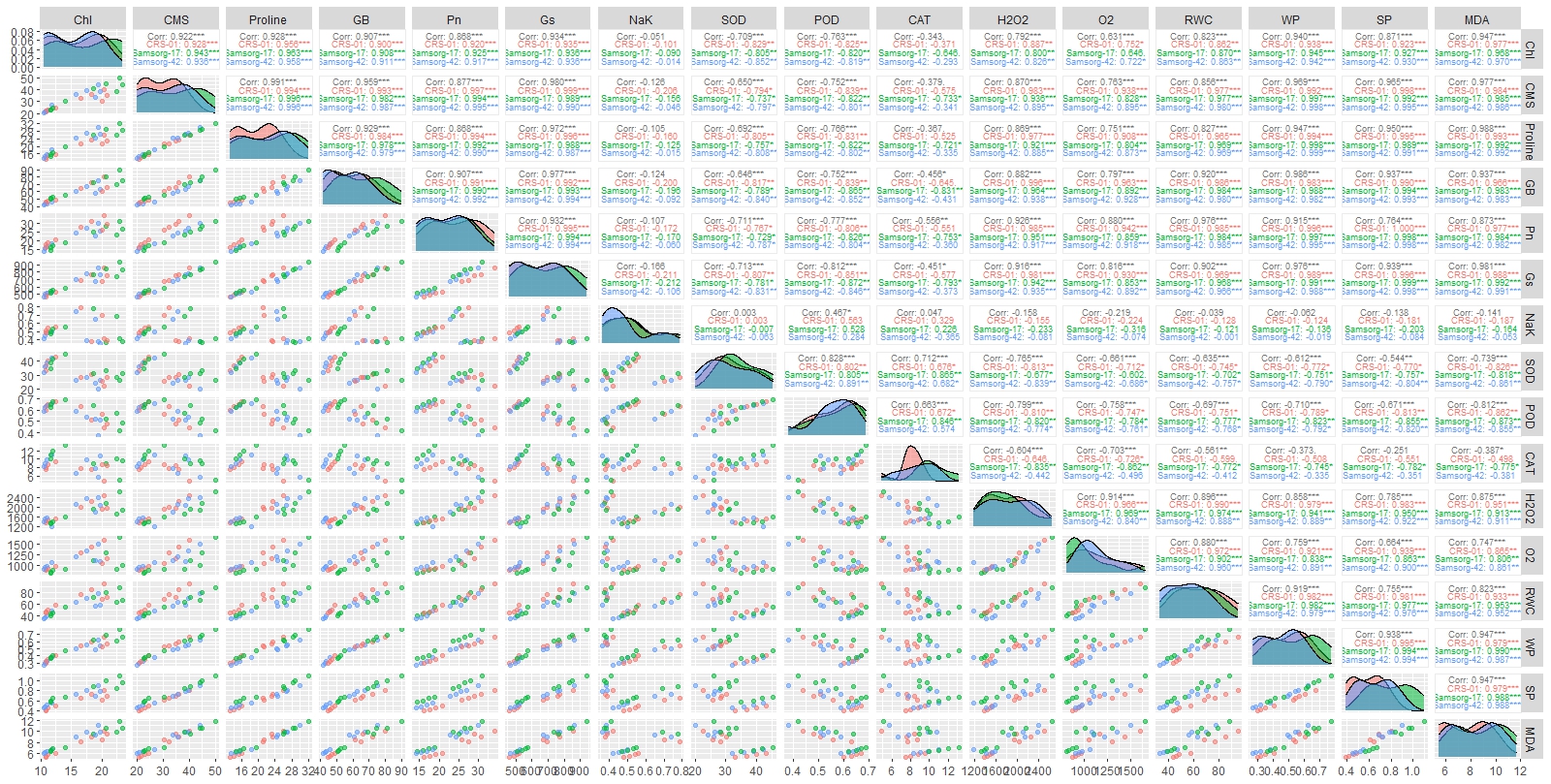

Supplement: Supplementary file 2 [file DataSheet1.zip › 18.jpeg]

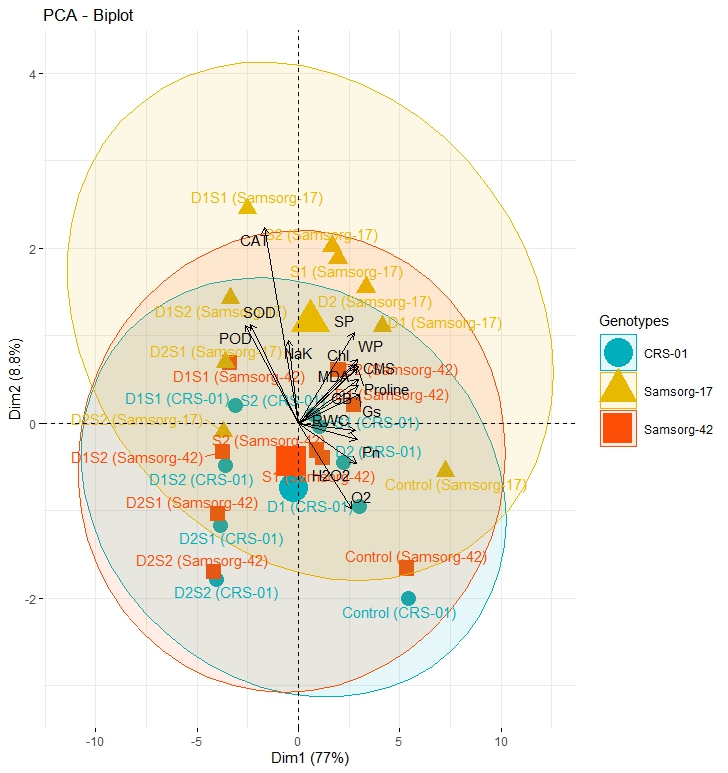

Supplement: Supplementary file 2 [file DataSheet1.zip › 19.jpeg]

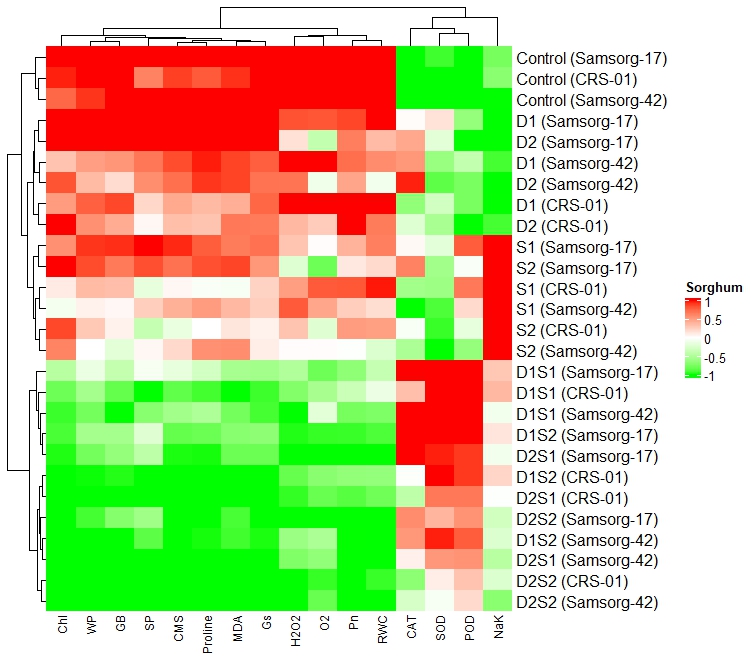

Supplement: Supplementary file 2 [file DataSheet1.zip › 20.jpeg]
