# Supplementary material for: Genotypic variability in stress responses of Sorghum bicolor under drought and salinity conditions
Source: Front Genet. 2025 Jan 8;15:1502900. doi: 10.3389/fgene.2024.1502900 (PMC11750996; doi:10.3389/fgene.2024.1502900)
Supplement: Supplementary file 3 [file Table2.docx]

Figure Captions

Figures 1-8 in zip:

**Figure 1a**. Effect of drought and salinity (individual and combined treatments) on catalase (CAT) activities of sorghum genotypes. The vertical bars represent means, and the error bars indicate statistical significance at p ≤ 0.05.

**Figure 1b.** Effect of drought and salinity (individual and combined treatments) on Superoxide dismutase (SOD) activities of sorghum genotypes. The vertical bars represent means, and the error bars indicate statistical significance at p ≤ 0.05.

**Figure 1c.** Effect of drought and salinity (individual and combined treatments) on Peroxidase (POD) activities of sorghum genotypes. The vertical bars represent means, and the error bars indicate statistical significance at p ≤ 0.05

**Figure 1d**. Effect of drought and salinity (individual and combined treatments) on Glycine betaine (GB) of sorghum genotypes. The vertical bars represent means, and the error bars indicate statistical significance at p ≤ 0.05

**Figure 1e**. Effect of drought and salinity (individual and combined treatments) on Superoxide anion radical content (O_2_^-^ ) of sorghum genotypes. The vertical bars represent means, and the error bars indicate statistical significance at p ≤ 0.05

**Figure 1f**. Effect of drought and salinity (individual and combined treatments) on hydrogen peroxide content (H_2_O_2_ ) of sorghum genotypes. The vertical bars represent means, and the error bars indicate statistical significance at p ≤ 0.05

**Figure 1g.** Effect of drought and salinity (individual and combined treatments) on Malondialdehyde (MDA) of sorghum genotypes. The vertical bars represent means, and the error bars indicate statistical significance at p ≤ 0.05. **Figure 1h.** Effect of drought and salinity (individual and combined treatments) on proline content of sorghum genotypes. The vertical bars represent means, and the error bars indicate statistical significance at p ≤ 0.05

Figures 9-16 in zip:

**Figure 2a**. Effect of drought and salinity (individual and combined treatments) on Chlorophyll content (Chl) of sorghum genotypes. The vertical bars represent means, and the error bars indicate statistical significance at p ≤ 0.05.

**Figure 2b**. Effect of drought and salinity (individual and combined treatments) on Cell membrance stability (CMS ) of sorghum genotypes. The vertical bars represent means, and the error bars indicate statistical significance at p ≤ 0.05

**Figure 2c**. Effect of drought and salinity (individual and combined treatments) on Stomatal conductance (Gs) of sorghum genotypes. The vertical bars represent means, and the error bars indicate statistical significance at p ≤ 0.05.

**Figure 2d.** Effect of drought and salinity (individual and combined treatments) on Na/K ratio of sorghum genotypes. The vertical bars represent means, and the error bars indicate statistical significance at p ≤ 0.05

**Figure 2e**. Effect of drought and salinity (individual and combined treatments) on Photosynthetic rate (Pn) of sorghum genotypes. The vertical bars represent means, and the error bars indicate statistical significance at p ≤ 0.05

**Figure 2f**. Effect of drought and salinity (individual and combined treatments) on Relative water content (RWC) of sorghum genotypes. The vertical bars represent means, and the error bars indicate statistical significance at p ≤ 0.05

**Figure 2g**. Effect of drought and salinity (individual and combined treatments) on Solute potential (SP) of sorghum genotypes. The vertical bars represent means, and the error bars indicate statistical significance at p ≤ 0.05. **Figure 2h.** Effect of drought and salinity (individual and combined treatments) on Water potential (WP) of sorghum genotypes. The vertical bars represent means, and the error bars indicate statistical significance at p ≤ 0.05

Figures 17-19 in zip:

**Figure. 4a**. The correlations among factors. The upper matrix shows the Pearson coefficients, and results were significant at *** p < 0.01, ** p < 0.05, or * p < 0.1 as marked. The red solid lines in the lower matrix show a smooth regression between the two factors.

**Figure 4b**. Pearson correlation matrices for the effects of individual and combined drought and salinity stresses on sorghum genotypes, with significance levels denoted as follows: * (p ≤ 0.1), ⁎⁎ (p ≤ 0.01), and ⁎⁎⁎ (p ≤ 0.001).

**Figure 4c**. PCA scatter plot showing the grouping of physiological and biochemical characteristics according to their resemblance and variation, particularly concerning various sorghum genotypes.

Figures 20 in zip

**Figure 5**. Cluster dendrogram heatmap depicting how physiological and biochemical traits respond in sorghum genotypes under stress conditions of individual and combined drought and salinity.

**Figure** 3 (gene figures)

**Figure 3a**. Relative expression of SbSOD1 gene in various sorghum genotypes under both individual and combined stresses of drought and salinity.

**Figure 3b**. Relative expression of SbAPX2 gene in various sorghum genotypes under both individual and combined stresses of drought and salinity.

**Figure 3c**. Relative expression of SbCAT3 gene in various sorghum genotypes under both individual and combined stresses of drought and salinity.

**Figure 3d.** Relative expression of SbHKT1;4 gene in various sorghum genotypes under both individual and combined stresses of drought and salinity.

**Figure 3e**. Relative expression of SbDREB2A gene in various sorghum genotypes under both individual and combined stresses of drought and salinity.

**Figure 3f**. Relative expression of SbDHN3 gene in various sorghum genotypes under both individual and combined stresses of drought and salinity.

**Figure. 3g.** Relative expression of SbPRP1 gene in various sorghum genotypes under both individual and combined stresses of drought and salinity.
