# Supplementary material for: Genotypic variability in stress responses of Sorghum bicolor under drought and salinity conditions
Source: Front Genet. 2025 Jan 8;15:1502900. doi: 10.3389/fgene.2024.1502900 (PMC11750996; doi:10.3389/fgene.2024.1502900)
Supplement: Supplementary file 4 [file Table1.docx]

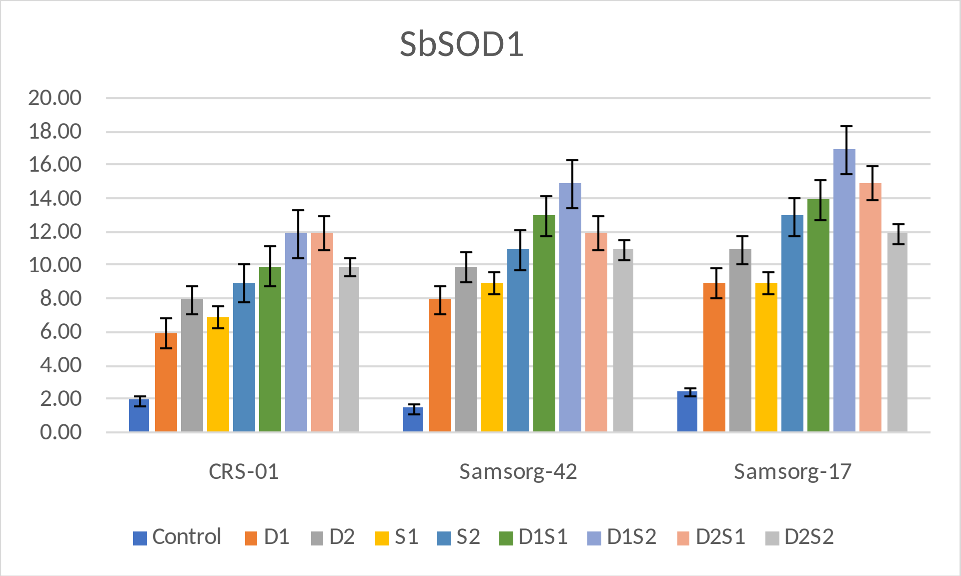
SbSOD1
20.00
18.00
16.00
14.00
12.00
10.00
8.00
6.00
4.00
2.00
0.00
CRS-01
Samsorg-42
Samsorg-17
Control
D1
D2
S1
S2
D1S1
D1S2
D2S1
D2S2

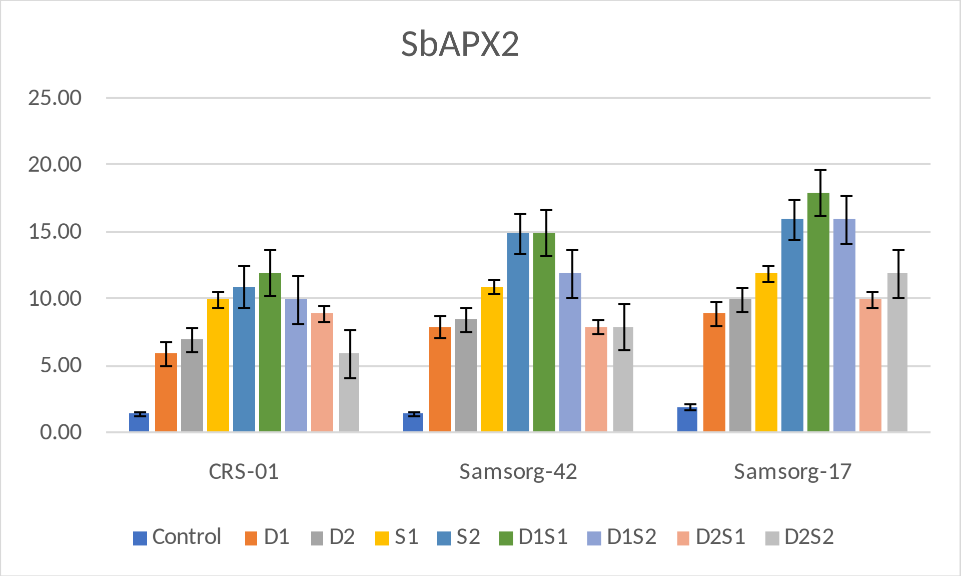
SbAPX2
25.00
20.00
15.00
10.00
5.00
0.00
CRS-01
Samsorg-42
Samsorg-17
Control
D1
D2
S1
S2
D1S1
D1S2
D2S1
D2S2

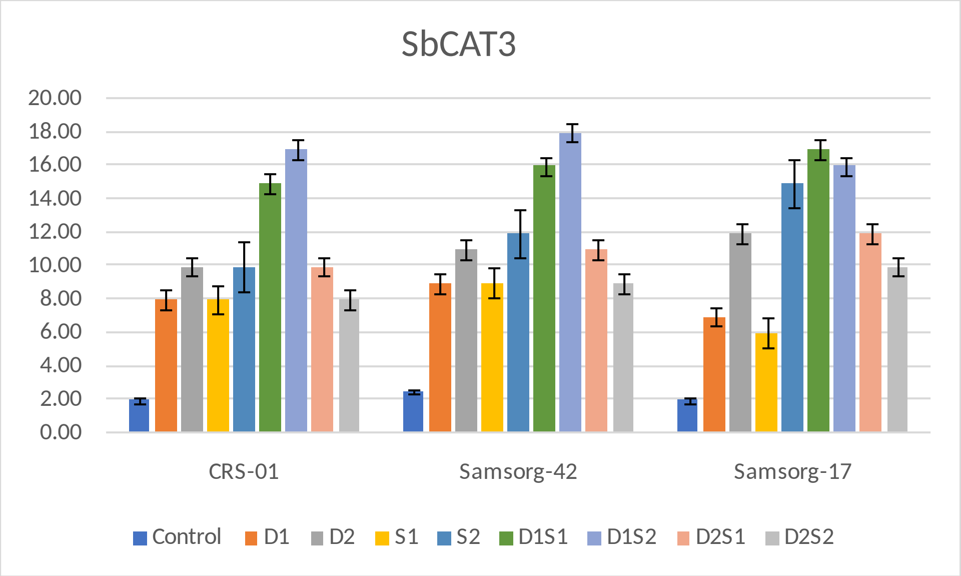
SbCAT3
20.00
18.00
16.00
14.00
12.00
10.00
8.00
6.00
4.00
2.00
0.00
CRS-01
Samsorg-42
Samsorg-17
Control
D1
D2
S1
S2
D1S1
D1S2
D2S1
D2S2

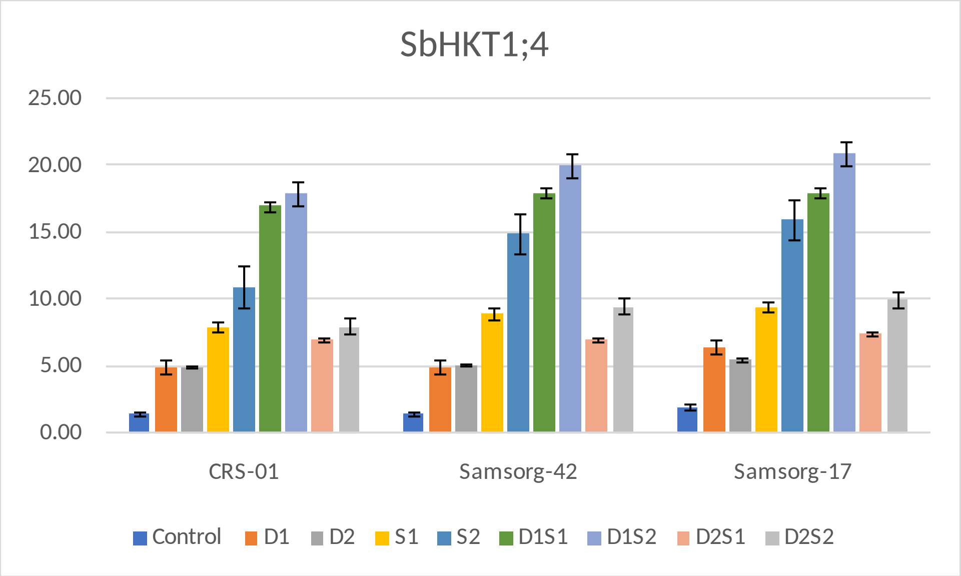
SbHKT1;4
25.00
20.00
15.00
10.00
5.00
0.00
CRS-01
Samsorg-42
Samsorg-17
Control
D1
D2
S1
S2
D1S1
D1S2
D2S1
D2S2

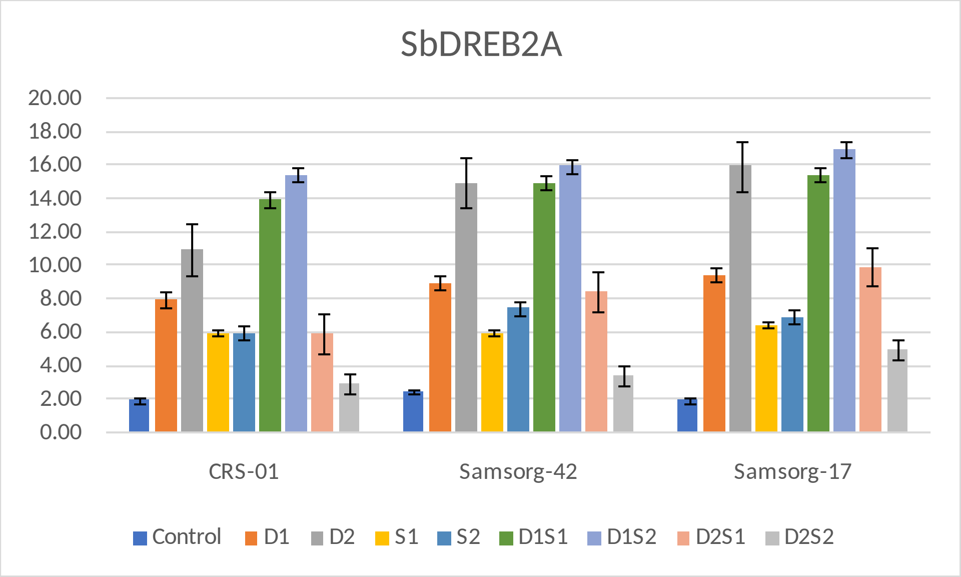
SbDREB2A
20.00
18.00
16.00
14.00
12.00
10.00
8.00
6.00
4.00
2.00
0.00
CRS-01
Samsorg-42
Samsorg-17
Control
D1
D2
S1
S2
D1S1
D1S2
D2S1
D2S2

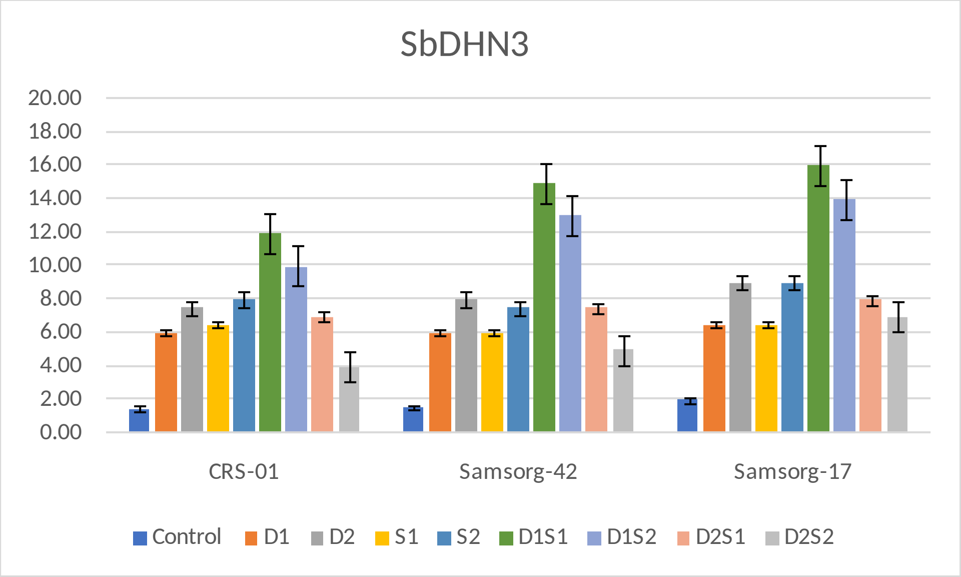
SbDHN3
20.00
18.00
16.00
14.00
12.00
10.00
8.00
6.00
4.00
2.00
0.00
CRS-01
Samsorg-42
Samsorg-17
Control
D1
D2
S1
S2
D1S1
D1S2
D2S1
D2S2

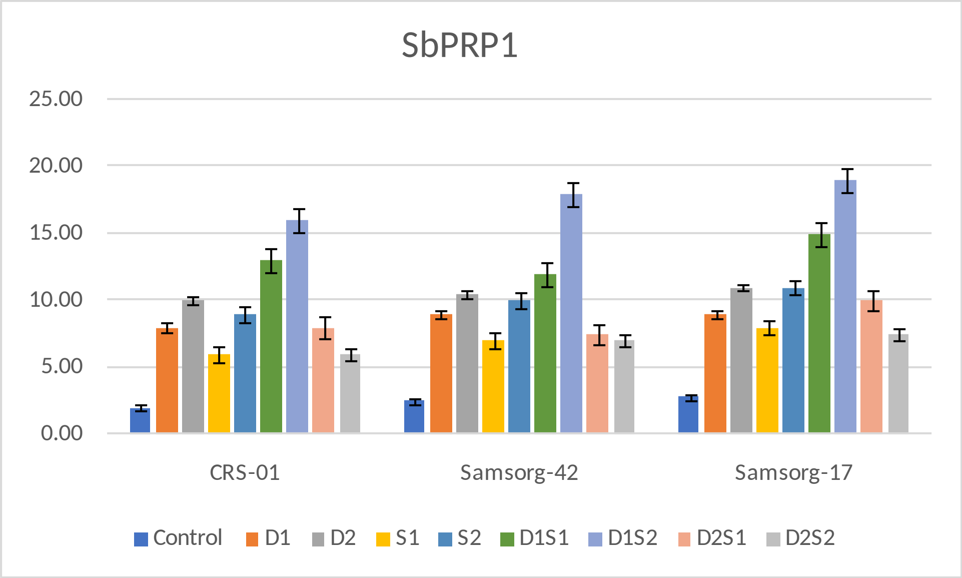
SbPRP1
25.00
20.00
15.00
10.00
5.00
0.00
CRS-01
Samsorg-42
Samsorg-17
Control
D1
D2
S1
S2
D1S1
D1S2
D2S1
D2S2
